# Zoledronate treatment duration is linked to bisphosphonate‐related osteonecrosis of the jaw prevalence in rice rats with generalized periodontitis

**DOI:** 10.1111/odi.13052

**Published:** 2019-02-19

**Authors:** Jonathan G. Messer, Jessica M. Jiron, Jorge L. Mendieta Calle, Evelyn J. Castillo, Ronnie Israel, Ean G. Phillips, Joshua F. Yarrow, Catherine Van Poznak, Lakshmyya Kesavalu, Donald B. Kimmel, J. Ignacio Aguirre

**Affiliations:** ^1^ Department of Physiological Sciences University of Florida Gainesville Florida; ^2^ Research Service VA Medical Center Gainesville Florida; ^3^ University of Michigan Ann Arbor Michigan; ^4^ Department of Periodontology and Oral Biology College of Dentistry Gainesville Florida

**Keywords:** anti‐resorptives, high sucrose‐casein diet, histopathology, preclinical, toxicology

## Abstract

**Objectives:**

To determine the extent that zoledronate (ZOL) dose and duration is associated with bisphosphonate‐related osteonecrosis of the jaw (BRONJ) prevalence in rice rats with generalized periodontitis (PD), characterize structural and tissue‐level features of BRONJ‐like lesions in this model, and examine the specific anti‐resorptive role of ZOL in BRONJ.

**Materials and Methods:**

Rice rats (*n* = 228) consumed high sucrose‐casein diet to enhance generalized PD. Groups of rats received 0, 8, 20, 50 or 125 µg/kg IV ZOL/4 weeks encompassing osteoporosis and oncology ZOL doses. Rats from each dose group (*n* = 9–16) were necropsied after 12, 18, 24 and 30 weeks of treatment. BRONJ‐like lesion prevalence and tissue‐level features were assessed grossly, histopathologically and by MicroCT. ZOL bone turnover effects were assessed by femoral peripheral quantitative computed tomography, serum bone turnover marker ELISAs and osteoclast immunolabelling.

**Results:**

Prevalence of BRONJ‐like lesions was significantly associated with (a) ZOL treatment duration, but plateaued at the lowest oncologic dose, and (b) there was a similar dose‐related plateau in the systemic anti‐resorptive effect of ZOL. ZOL and BRONJ‐like lesions also altered the structural and tissue‐level features of the jaw.

**Conclusion:**

The relationship between BRONJ‐like lesion prevalence and ZOL dose and duration varies depending on the co‐ or pre‐existing oral risk factor. At clinically relevant doses of ZOL, BRONJ‐like lesions are associated with anti‐resorptive activity.

## 1 INTRODUCTION

Medication‐related osteonecrosis of the jaw (MRONJ) is a potentially severe adverse event characterized by persistent exposure of necrotic bone in the jaw (Ruggiero et al., 2014) in patients treated with potent anti‐resorptives (pARs) [e.g., nitrogen‐containing bisphosphonates, N‐BPs (Marx, 2003; Ruggiero et al., 2014), and receptor activator of NFκB ligand antibodies (Stopeck et al., 2010; Van den Wyngaert, Wouters, Huizing, & Vermorken, 2011)]. More recently, MRONJ has been observed in patients with cancer taking anti‐angiogenic medication (e.g., vascular endothelial growth factor antibodies) (Carlson & Schlott, 2014; Eleutherakis‐Papaiakovou & Bamias, 2017; Fusco, Santini, Armento, Tonini, & Campisi, 2016; Khan et al., 2017; Ramirez, Lopez‐Pintor, Casanas, Arriba, & Hernandez, 2015; Voss, Poxleitner, Schmelzeisen, Stricker, & Semper‐Hogg, 2017). In ONJ cases in which the only systemically administered medication is an N‐BP, the term bisphosphonate‐related osteonecrosis of the jaw (BRONJ) may also be used.

Systemic administration of these medications is specified in the clinical definition of MRONJ as a requirement for a formal diagnosis (Ruggiero et al., 2014). However, local factors in the oral cavity, despite being absent from the formal definition of MRONJ, also increase risk and play a crucial role in the disease process (Aghaloo, Hazboun, & Tetradis, 2015; Aljohani et al., 2017; Carlson & Schlott, 2014; Eleutherakis‐Papaiakovou & Bamias, 2017; Filleul, Crompot, & Saussez, 2010; Hamadeh, Ngwa, & Gong, 2015; Khan et al., 2017; Marx, 2003; Otto et al., 2012; Voss et al., 2017; Yoneda et al., 2017).

Discrete oral procedures involving the dentoalveolar bone (e.g., tooth extraction) are considered the most common local oral factor associated with MRONJ and are estimated to occur in 52%–86% of MRONJ cases (Aghaloo et al., 2015; Aljohani et al., 2017; Carlson & Schlott, 2014; Eleutherakis‐Papaiakovou & Bamias, 2017; Filleul et al., 2010; Hamadeh et al., 2015; Khan et al., 2017; Marx, 2003; Otto et al., 2012; Ruggiero et al., 2014; Voss et al., 2017; Yoneda et al., 2017). Delaying the start of risk factor medications until surgical sites have healed, and ensuring appropriate perioperative management, including antibiotic coverage, are often effective means to prevent MRONJ associated with dentoalveolar surgery (Dimopoulos et al., 2009; Hoefert & Eufinger, 2011; Montefusco et al., 2008; Ripamonti et al., 2009; Vandone et al., 2012). However, periodontitis (PD) and periapical infection (Aghaloo et al., ; Aguirre, Akhter, Kimmel, Pingel, Williams et al., 2012; Li et al., 2016; Song et al., 2016) have also been identified as important risk factors for BRONJ/MRONJ in patients without recent dentoalveolar surgery (Carlson & Schlott, 2014; Eleutherakis‐Papaiakovou & Bamias, 2017; Khan et al., 2017; Marx, 2003; Voss et al., 2017). While oral diseases involving the periodontal tissues appear to contribute to MRONJ directly, they may also contribute to cases related to dentoalveolar surgery, since teeth that require extraction often have some degree of acute or chronic infection (e.g., infection from caries, periodontal disease). Cases in which MRONJ arises without a recent dentoalveolar surgery may be more difficult to anticipate and treat, especially if these conditions are not fully resolved before beginning pAR treatment, or if they arise after pAR treatment has started. Therefore, studies that investigate the role of specific oral inflammatory diseases as local oral risk factors for MRONJ are important to develop a comprehensive understanding of the pathophysiology of the disease, and contribute evidence that might be used to develop preventative or therapeutic strategies.

Rice rats (*Oryzomys palustris*) develop two distinctive types of PD that require no surgical or local mechanical intervention: (a) *a generalized form*, which affects both maxillae and mandibles, and is accelerated by a high sucrose‐casein diet (HSC) (Aguirre, Akhter, Kimmel, Pingel, Xia et al., 2012; Aguirre et al., 2016; Gotcher & Jee, 1981; Gupta & Shaw, ); and (b) *a localized form* (food impaction localized PD, FILP), which overwhelmingly affects the maxillary interdental space between molar (M) 2 and M3 and is found in rice rats fed standard rodent chow (STD) (Messer et al., 2017). Importantly, rice rats fed HSC diet do not develop FILP lesions. Therefore, two different PD models can be generated in rice rats by dietary modification alone. The pelleted HSC diet that accelerates generalized PD is based on the powdered Harvard high sucrose 700 and the ration 100 diets used in previous studies to accelerate the onset of PD in rice rats (Auskaps, Gupta, & Shaw, 1957; Gotcher & Jee, 1981; Gupta & Shaw, ; Ryder, 1980). The reason the pelleted HSC diet does not produce FILP (Aguirre, Akhter, Kimmel, Pingel, Williams et al., 2012; Aguirre, Akhter, Kimmel, Pingel, Xia et al., 2012) is possibly due to the low content (3%) of insoluble fibre provided as powdered cellulose, which is in contrast to STD rodent chow (irradiated diet no. 7912, Teklad LM‐485 Rodent Diet; Envigo, Tampa, FL), which contains 13.7% neutral detergent fibre (NDF) and 4.6% of crude fibre. These fibrous components of the STD chow diet appear to contribute to the initiation and persistence of FILP lesions.

The defining feature of the FILP model is direct injury to soft tissue in the maxillary interdental M2‐M3 space by persistent presence of impacted plant fibre and cellular debris. Food impaction around teeth, dental implants, or embrasures in humans may also result in peri‐implantitis or peri‐coronitis (Du, Gao, Qi, Liu, & Lin, 2010), and is an important risk factor for localized PD in humans (Matthews & Tabesh, 2004; Nunn, 2003). Conversely, clinical and histopathologic examination of generalized PD in the rice rat shows marked accumulation of microbial plaque on molar surfaces and inflammation of periodontal tissues. This type appears to resemble human PD, in which biofilms are an important mediator and contributing factor to the pathophysiology of the disease (Genco & Borgnakke, 2013; Offenbacher, 1996; Page, Offenbacher, Schroeder, Seymour, & Kornman, 1997; Salvi, Lawrence, Offenbacher, & Beck, 1997).

Rice rats with either type of PD, and without dentoalveolar surgery, develop BRONJ‐like lesions when simultaneously exposed to clinically relevant doses of the systemic pAR, zoledronate (ZOL) (Aguirre, Akhter, Kimmel, Pingel, Williams et al., 2012; Messer et al., 2018). Importantly, the anatomical distribution of BRONJ‐like lesions appears to be associated with the anatomical location of the PD in individual rats. Specifically, rice rats fed STD diet and injected with ZOL developed BRONJ‐like lesions primarily at the same location as the FILP lesions, the M2M3 maxillary interdental space (Messer et al., 2018), whereas rats fed HSC diet and injected with oncologic ZOL developed BRONJ‐like lesions equally in the four jaw quadrants (Aguirre, Akhter, Kimmel, Pingel, Williams et al., 2012). These findings suggest that rats given clinically relevant doses of ZOL are more likely to develop BRONJ‐like lesions in quadrants that are also directly affected by oral inflammation.

In humans, various local oral risk factors (e.g., oral infections, surgical/trauma events and inflammatory disease), when combined with systemic risk factor medications, can result in MRONJ. However, it is not clear if different local oral risk factors result in distinctive patterns in the relationship between MRONJ prevalence and dose and duration of systemic risk factor medications. The rice rat provides a unique opportunity to explore this hypothesis given the distinctive types of PD that exist in this species. Previous findings demonstrated that BRONJ‐like lesions occur in rice rats with localized PD (FILP) in a positive dose‐dependent manner, but ZOL duration did not have a significant effect on the prevalence of BRONJ‐like lesions, which plateaued between 18 and 30 weeks of treatment (Messer et al., 2018). Importantly, 50%–75% of vehicle‐treated STD diet rats show FILP‐induced mucosal injury by age 16 weeks. This finding is in direct contrast to the age‐related progression of generalized PD which does not become moderate to severe in a majority of rats until later, at age 22 weeks (Aguirre, Akhter, Kimmel, Pingel, Xia et al., 2012). These findings suggest that the effect of ZOL dose and duration on BRONJ‐like lesion prevalence in rice rats with generalized PD could differ from that in rice rats with localized PD. Examining how different oral risk factors work alongside systemic medications may provide important data that improves the current understanding of how different periodontal risk factors interact with systemic medications to initiate MRONJ.

The primary purposes of this study were to: (a) define the relationship between ZOL dose and duration and prevalence of BRONJ‐like lesions in rice rats with generalized PD, (b) characterize the overall structure and tissue‐level features of alveolar bone in ZOL‐treated rice rats with and without BRONJ‐like lesions by MicroCT and histologic techniques and (c) examine the association between the anti‐resorptive effect of ZOL at clinically relevant doses and the presence of BRONJ‐like lesions.

We hypothesize that: (a) increased dose and duration of ZOL will increase the prevalence of BRONJ‐like lesions in rice rats with generalized PD in a pattern that is distinctive from previous findings in rats with localized PD; (b) ZOL and BRONJ‐like lesions will alter the structure and tissue‐level features of the jaw; and (c) ZOL dose will be associated with reduced osteoclast number in the jaw and suppressed systemic bone resorption. Testing the first hypothesis will involve using data from a previously reported experiment (Messer et al., 2018) conducted simultaneously to this one.

## 2 MATERIALS AND METHODS

### 2.1 Animal care

A monogamous continuous‐breeding system was used to generate the experimental rice rats. All rats were housed (2–5 per cage) in static filter top cages (area: 143 in^2^) with pine shavings and continuous access to food and water. The housing rooms were maintained at 20–26ºC with humidity of 30%–70% and a 12:12 hr light:dark cycle, or 14:10 hr cycle for breeders (Aguirre et al., 2015). Experimental animals ate pelleted, semi‐purified HSC diet (TestDiet 5SXA AIN‐93G w/no Complex Carbohydrates; Smelt Feed and Pet Supply, Tampa, FL, USA), which can be fed for up to 30 weeks without inducing significant metabolic abnormalities (Aguirre et al., 2016). This diet is based on the formula of the powdered Harvard high sucrose 700 diet and the ration 100 diet previously used to accelerate generalized PD in rice rats (Auskaps et al., 1957; Gotcher & Jee, 1981; Gupta & Shaw, ; Ryder, 1980). Breeder rats ate rodent breeder chow (Envigo Teklad, irradiated 2919). Body weight (BW) was measured bi‐weekly. If rats showed BW loss and/or reduced body condition score (BCS), saline was administered subcutaneously and gel diet was offered (Ullman‐Cullere & Foltz, 1999). The Animal Care Services resource at the University of Florida (UF) is an AAALAC‐accredited programme. The breeding (IACUC#201408452) and experimental (IACUC#201408453) protocols were approved by the UF Institutional Animal Care and Use Committee (IACUC), and all experiments were compliant with the ARRIVE guidelines.

### 2.2 Study design

**Figure 1:**
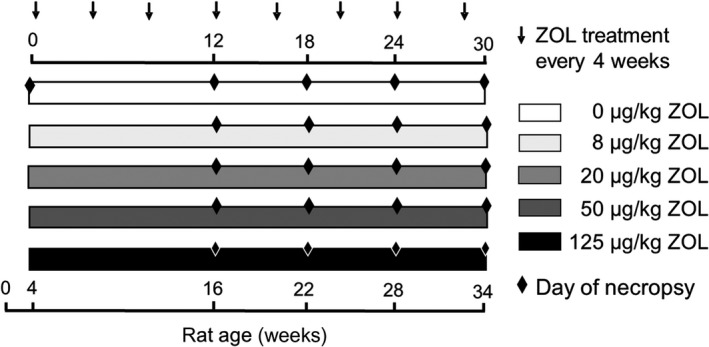
Experimental design. Rats were weaned at age 4 weeks, placed on high sucrose‐casein diet and randomized into dosing groups. Starting at age 4 weeks, rats received tail vein injections of 0, 8, 20, 50 or 125 µg/kg zoledronate (ZOL) q4wks until necropsy. One group of rats was necropsied at age 4 weeks (*n* = 12). Groups of rats (*n* = 9–16) within each dose level were necropsied at 12, 18, 24 or 30 weeks of ZOL treatment

Two hundred and twenty‐eight clinically normal, female weanling rice rats (BW ≥ 30 g; BCS ≥ 3.0) were BW‐randomized into a baseline group (*n* = 12; age, 4 weeks) or one of five dose groups (Figure 1), with efforts made to assign littermates into different dose groups. A previous study showed that both male and female rice rats given oncologic doses of ZOL develop BRONJ (Aguirre, Akhter, Kimmel, Pingel, Williams et al., 2012). Only females were used in the current study to avoid variation in measurements due to sexual dimorphism (Aguirre et al., 2015). All groups were fed pelleted HSC diet and received intravenous (IV) injections of 0 (*n* = 42), 8 (*n* = 44), 20 (*n* = 44), 50 (*n* = 40), or 125 µg/kg BW of ZOL (*n* = 46) every four weeks (q4wk), starting at 4 weeks of age. After 12, 18, 24 and 30 weeks of treatment, duration subgroups (*n* = 9–16) from each of the five dose groups were necropsied (Supporting Information Table S1 and Figure S1). The minimum sample size (*n* = 9) was determined by power calculation (see Statistical Analysis, Section 2.11). Animals were added to later time points based on a 10% censor rate to account for rats removed from the study before reaching scheduled time points, based on attrition rates from previous long‐term rice rat studies. To compose each duration subgroup, rats with progressive BW loss that reached ≥15% were chosen to comply with humane endpoints specified in the IACUC protocol. The remaining rats for each duration subgroup were selected by BW randomization. No more than 30% of each dose/duration subgroup was composed of rats that met the BW loss endpoint criteria for euthanasia.

### 2.3 ZOL dose

The dosing strategy has been previously described (Messer et al., 2018). Briefly, we used 8 μg/kg IV q4wk as the rat equivalent of the human osteoporosis (OP) dose. The dose gradient in the current study includes 0, 8, 20, 50 and 125 µg/kg, which encompasses both the OP and a range of oncologic ZOL doses. For each dose concentration, ZOL was dissolved in normal sterile saline (pH 7.2). ZOL was injected into the tail vein of rats placed in rodent restrainers at the rate of 0.4 ml/100 g BW. ZOL was kindly provided by Novartis Pharma AG (Basel, Switzerland).

### 2.4 Euthanasia and tissue collection

Rats were euthanized by CO_2_ followed by cardiac puncture to withdraw about 1.5 ml of whole blood. Cervical dislocation was used as a secondary confirmatory method of euthanasia. Maxillae and mandibles were excised as before (Messer et al., 2018). High‐resolution (HR) photographs from the medial aspect of each hemi‐mandible (Supporting Information Figure S2a) and ventral aspect of each hemi‐maxillae (Supporting Information Figure S2b) were taken with a digital camera (Canon EOS 6D; Tokyo, Japan) attached to a macro lens (Canon EF 100 mm 1:2.8; Tokyo, Japan). Jaw quadrants were fixed at 4°C in freshly prepared 4% paraformaldehyde, then transferred to 70% ethanol after 48 hr.

### 2.5 Gross analysis of oral lesions

Jaw quadrant photographs were independently assessed by blinded observers (JGM, DBK, JIA). Quadrants were assigned a *Gross Quadrant Grade (GQG)* that reflected the severity of its gross lesions (Table 1 **)** (Messer et al., 2018). Photographs of representative maxillae and mandibles representing each *GQG* are shown in Supporting Information Figure S2. When at least one quadrant in a rat had an area of exposed bone, the rat was considered positive for a gross BRONJ‐like lesion. Neither pocket depth nor clinical attachment level by probing were evaluated. Photographs that did not receive the same *GQG* from all three independent observers were re‐examined by all three observers together, and a *GQG* was assigned when consensus was reached.

**Table 1:** Criteria for Gross Quadrant Score (*GQG*)

Score	Degree	Description
0	None	Normal gingival contours
1	Slight	Minimal plaque, minimal gingival recession
2	Mild	Plaque, recession/ulceration of gingiva with limited extension towards lingual plate or palatal midline; inflammation/swelling/redness at margin of lesion
3	Moderate	Plaque, recession/ulceration gingival extending into lingual mucosa towards midline in maxilla or onto lingual mucosa in mandible; inflammation/swelling/redness at margin of lesion; possible involvement of buccal mucosa, possible tooth migration
4	Severe	Profound plaque accumulation, extensive gingival recession/ulceration involving all three molars and extending into lingual mucosa towards the midline and onto the buccal mucosa; gingival inflammation/redness; loss of alveolar bone with furcation exposure, tooth migration or tooth loss
BRONJ		Lesion of *GQG = *3 or 4 with *exposure of alveolar or palatal bone*

Note

See Supporting Information Figure 2 for corresponding photographs.

### 2.6 Histopathologic assessment of quadrants

#### 2.6.1 Specimen selection for histopathologic BRONJ‐like lesion analysis

Histopathologic confirmation of BRONJ‐like cases was undertaken in rats that were: (a) diagnosed with gross BRONJ‐like lesions; (b) not diagnosed with gross BRONJ‐like lesions, but had quadrants with oral lesions of *GQG* ≥2; and (c) not diagnosed with gross BRONJ‐like lesions, but experienced prenecropsy BW loss (see Supporting Information Figure S1). Quadrants with *GQG* ≥ 2 were selected because these quadrants displayed gross gingival recession and ulceration. For rats with BW loss, all quadrants were analysed because data from a previous study showed that rice rats with BRONJ‐like lesions had BW loss compared to rice rats without these lesions (Messer et al., 2018). In the current study, 37% (80/218) of rats necropsied at weeks 12–30 exhibited BW loss. 92% of rats with gross BRONJ‐like lesions (22/24) and 73% (22/30) of rats with BRONJ‐like lesions diagnosed only using histopathology (without prior gross diagnosis) had BW loss.

#### 2.6.2 Specimen selection for PD analysis

Right maxillae and mandibles from all ZOL 0 rats, and right maxillae from baseline rats, regardless of *GQG*, were examined to determine periodontal status and assigned a PD Score (Table 2). When right quadrants were not available, left quadrants were used.

**Table 2:** PD score (inflammation scoring system)

Score	Degree	Description
0	Absence	None
1	Slight	Gingivitis: slight hyperplasia of GE, intraepithelial inflammatory cell infiltration. Bacterial plaque accumulation. Normal LP, PDL and ABC
2	Mild	Gingival hyperplasia, inflammatory cell infiltration of the GE and LP. Bacterial plaque accumulation. Normal PDL and ABC
3	Moderate	Erosion/ulceration and hyperplasia of the GE and marked bacterial plaque accumulation. Moderate inflammatory cell infiltration of LP, disruption of PDL, migration of the junctional epithelium and mild ABC resorption
4	Severe	Ulceration/hyperplasia of the GE with marked bacterial plaque accumulation. Severe inflammatory cell infiltration of LP, disruption of PDL, migration of the junctional epithelium and marked ABC resorption

Note

ABC, alveolar bone crest; GE, gingival epithelium; LP, lamina propria; PDL, periodontal ligament.

### 2.7 Histological preparation of jaw quadrants

Maxillae and mandibles selected for sectioning were decalcified in 5% formic acid under gentle agitation for 4 weeks, with three changes per wk. Decalcified jaw quadrants were trimmed, paraffin‐embedded, and sectioned at 5 µm thickness in the mesiodistal plane from the lingual to buccal aspect of each quadrant (Leica/Jung 2265 and Accu‐Cut SRM 200 Sakura microtomes, Sakura Finetek Europe B.V, Zoeterwoude, The Netherlands). Maxillae and mandibles were serially sectioned at five or six levels, starting approximately 1 mm lateral to the midline of the palate or at the lingual plate, respectively. Levels were separated by approximately 250 µm, allowing thorough examination of tissues at the lingual surface (one or two levels), around the molars (three levels), and at the buccal surface (one level). Sections were H&E stained and cover slipped.

#### 2.7.1 Histopathologic definition of BRONJ‐like lesions and quantification of necrotic bone

Histopathologic BRONJ‐like lesions were defined as bone that was both exposed and necrotic in at least one level within a quadrant. Exposed bone was identified by a lack of overlying gingival epithelium, lamina propria, and periodontal ligament (PDL). Necrotic bone was identified using (a) a pattern recognition approach as previously described (Franco‐Pretto, Pacheco, Moreno, Messa, & Gnecco, 2014; Hellstein, 2014; Yang et al., 2009; Zheng et al., 2018), and (b) by specific criteria used in previous preclinical studies of BRONJ that define necrotic bone as bone matrix containing ≥10 adjacent lacunae that were empty, or had pyknotic osteocyte nuclei or cellular debris (Kuroshima & Yamashita, 2013; Yamashita, Koi, Yang, & McCauley, 2011). All levels were surveyed independently by two observers (JGM and JIA). When diagnoses differed, agreement was reached after reviewing slides together. A rat with at least one quadrant that met the definition above was considered positive for BRONJ.

To quantify bone necrosis, the number of lacunae that were empty or contained pyknotic nuclei was counted. Data collection was performed at 200× magnification in sections within a 0.15–0.25 mm^2^ region of interest (ROI) that contained exposed bone matrix in a BRONJ‐like lesion, or an area of bone with periodontal tissue destruction and inflammation in FILP lesions with *GQG* ≥ 1. Total bone area (B.Ar, mm^2^), total number of osteocyte lacunae, and number of empty osteocyte lacunae were measured. The percentage of empty lacunae, and the number of empty lacunae per B.Ar (#/mm^2^), were calculated.

#### 2.7.2 Histopathologic characterization of PD

**Figure 2:**
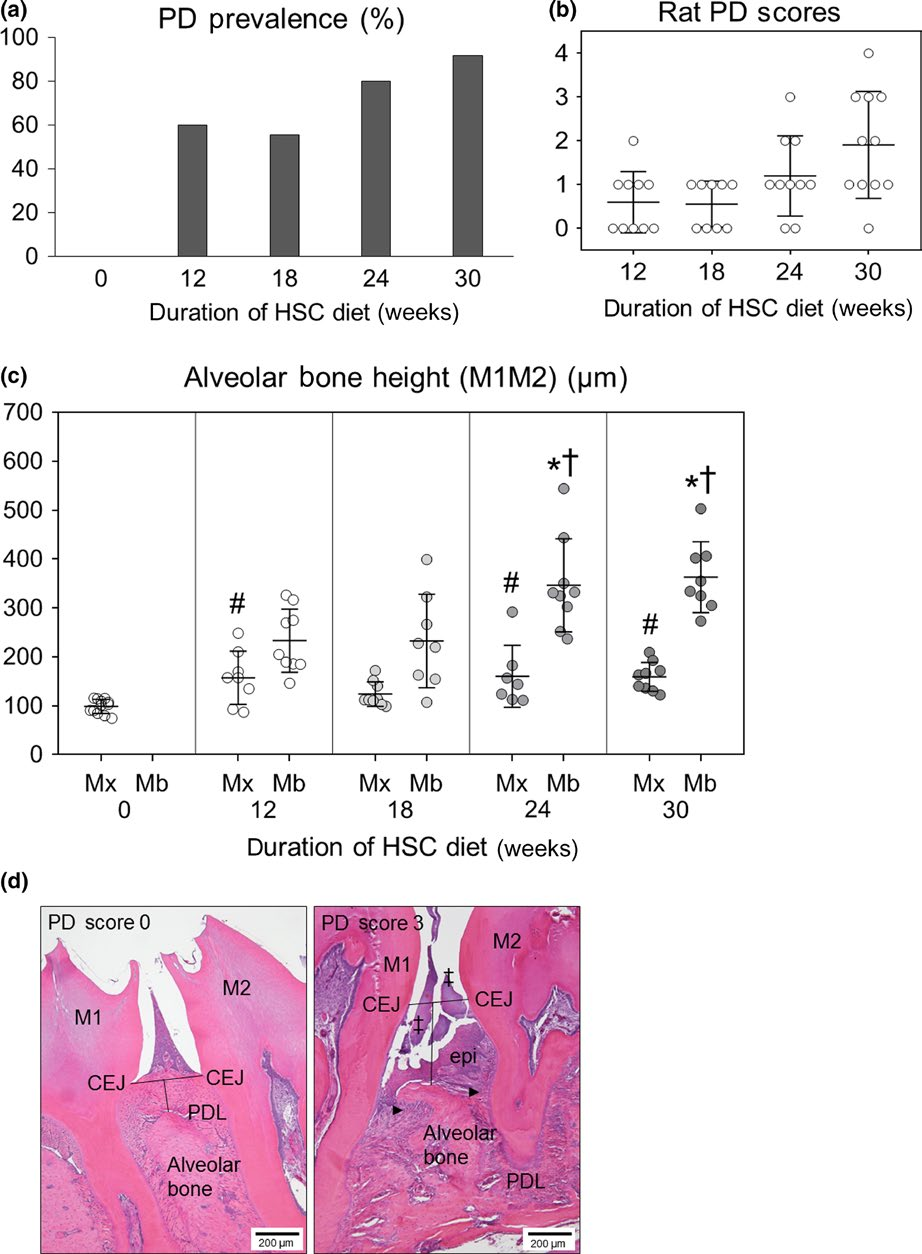
Periodontitis (PD) status of control (ZOL 0) rats after weaning onto high sucrose‐casein (HSC) diet. (a) PD prevalence was 0% at 0 weeks and peaked at 92% by 30 weeks. (b) Dot density plot of PD Score for each rat. There was a positive correlation between PD Score and time on HSC diet (*R* = 0.496, *p* = 0.00124). (c) Alveolar bone height (ABH), measured as the distance between the cementoenamel junction (CEJ) to the alveolar bone crest, in mandibles was significantly lower after 24 and 30 weeks than at 12 and 18 weeks; ABH in maxillae was significantly lower after 12, 24 and 30 weeks compared to baseline; * indicates *p < *0.05 compared to 12 weeks in mandibles, † indicates *p < *0.05 compared to 18 weeks in mandibles, # indicates *p < *0.05 compared to 0 weeks in maxillae. Mean ± *SD*. (d) Comparison of mandibular interdental regions (M1M2) of PD Score 0 and PD Score 3; PD Score 3 is characterized by plaque accumulation (‡), epithelial (epi) hyperplasia, junctional epithelial apical migration, inflammatory infiltrate (black arrow head) in periodontal ligament (PDL) and alveolar bone loss

Sections were assessed for PD Score (Table 2) and alveolar bone loss as previously described (Aguirre, Akhter, Kimmel, Pingel, Xia et al., 2012). A quadrant with PD Score ≥1 was considered positive for PD. A rat with at least one quadrant with PD Score ≥1 was considered positive for PD. The rat PD Score is the highest quadrant PD score for that animal. Alveolar bone height (ABH) was assessed in both maxillae and mandibles by measuring the distance between the cementoenamel junction (CEJ) and the crest of the alveolar bone, as previously described (Figure 2d) (Aguirre, Akhter, Kimmel, Pingel, Williams et al., 2012; Aguirre et al., 2016). Measurements were made in one section from the lingual aspect of the M1M2 interdental space. A larger ABH value indicates greater loss of alveolar bone. Measurements were made in blinded, randomized order (JGM). ABH in baseline rats was only measured in maxillae.

#### 2.7.3 Osteoclast quantification

To assess the local effect of ZOL, PD, and BRONJ on osteoclasts in the jaws, osteoclasts were analysed on the surfaces of maxillary alveolar bone with PD (PD Score ≥1) in rats treated with ZOL 0 (*n* = 11) or ZOL 8–125 (*n* = 4–12), ZOL‐treated rats with BRONJ (*n* = 10), or ZOL 0 rats with no PD (*n* = 12). Osteoclasts were identified in sections of paraffin‐embedded maxillae using immunohistochemical staining of tartrate‐resistant acid phosphatase 5b (TRAP), a protein specifically expressed in active osteoclasts. Slides were deparaffinized and endogenous peroxidases were blocked with 3% hydrogen peroxide in methanol, and then re‐hydrated. Slides were blocked with 2% goat serum in Tris‐buffered saline with 1% Tween and incubated overnight with primary rabbit anti‐TRAP at 1:600 dilution (Abcam; Cambridge, United Kingdom). Vectastain ABC Rabbit IgG kit (Vector Labs; Burlingame, CA, USA) and ImmPACT DAB peroxidase substrate kit (Vector Labs) were used to detect signal using manufacturers’ instructions. Tissue was counterstained with SelecTech haematoxylin 560 (Leica; Wetzlar, Germany). Negative control tissues were processed the same, but were not incubated with primary antibody. TRAP‐positive cells were counted on alveolar bone surfaces and bone perimeter (B.Pm, mm) was measured using Osteomeasure software (Osteometrics Corporation; Decatur, GA, USA) in sections from regions that included interdental M1M2 and M2M3 and was proximal to the lingual aspect of the molars. The number of TRAP+ cells per millimetre of B.Pm was calculated (#/mm).

### 2.8 MicroCT

A subset of 40 mandibles underwent MicroCT scanning prior to decalcification to characterize the effect of ZOL and BRONJ on calcified tissues of the jaw. Mandibles (*n* = 10/group) from rats treated with 0 or 20–125 µg/kg BW ZOL were grouped according to: (a) gross BRONJ; (b) *GQG* 1–4 with histopathologic BRONJ; (c) *GQG* 1–4 without gross BRONJ; and (d) *GQG = *0 (ZOL 0). Two‐dimensional (2D) bone morphology of the mandible was evaluated by 10 µm voxel size MicroCT (Bruker Skyscan 1172, Kontich, Belgium) as described (Beck et al., 2014; Yarrow, Conover et al., 2014; Yarrow, Ye et al., 2014) and recommended (Bouxsein et al., 2010). Images were acquired using the following parameters: 80 kVP/120 µA, 0.5 mm aluminium filter, 2k camera resolution, 10 µm voxel size, 0.5° rotation step, and 360° tomographic rotation.

Two analyses were completed. First, 2D images were aligned in the mesiodistal plane with all molars simultaneously visible for qualitative image analysis. Second, 2D images from the mesial aspect of M2 were aligned in the buccolingual plane and the width of the alveolar ridge was measured (Figure 9d). Mandibles from ZOL 0 rats were compared to rats treated with oncologic ZOL (20–125). To consistently measure the same area in all mandibles, the slice through the apical foramen of the mesial root of M2 was selected. Then, the vertical length of M2 between the CEJ and apex of the mesial root was measured. The buccolingual width of the alveolar ridge was then measured perpendicular to the M2 mesial root at 30% (coronal width) and 60% (apical width) the total length of the CEJ‐apex distance (ImageJ Software, NIH). After scanning, quadrants underwent histopathologic analysis (See Sections 2.6 and 2.7).

### 2.9 Peripheral quantitative computed tomography

Peripheral quantitative computed tomography (pQCT) analysis of the femur was conducted to verify the anti‐resorptive efficacy of ZOL, and act as a pharmacodynamic marker of anti‐resorptive activity that can be compared to BRONJ prevalence. At necropsy, right femurs were disarticulated from the acetabulum and separated intact from the tibia, wrapped in saline‐soaked gauze and stored frozen at −20°C until analysis. One‐mm‐thick cross sections were scanned using a Stratec XCT Research M instrument (version 5.40, Norland Medical Systems; Fort Atkinson, WI) with manufacturer's software. Sites of interest were 5 mm proximal to the distal end of the femur (distal metaphysis), and the longitudinal midpoint of the femur (mid‐diaphysis). Volumetric bone mineral content (vBMC, mg), volumetric bone mineral density (vBMD, mg/cm^3^) and cortical area (mm^2^) were determined for total bone (cancellous and cortical bone at the metaphysis, and cortical bone at the mid‐diaphysis), as previously described (Ke, Qi, Chidsey‐Frink, Crawford, & Thompson, 2001).

### 2.10 Serum ELISA

Serum concentration of P1NP, a bone formation marker and tartrate‐resistant acid phosphatase 5b (TRAcP 5b), a marker of osteoclast number, were measured to characterize the systemic effects of ZOL on the skeleton. After cardiac puncture, blood was allowed to clot at room temperature and centrifuged, and serum was collected and stored at −20°C. Rat/Mouse P1NP EIA and the MouseTRAP kits were used, per manufacturer's instructions (Immunodiagnostic Systems; Tyne & Wear, UK), in serum collected from rats treated for 24 weeks.

### 2.11 Statistical analysis

Chi‐square test was used to determine the relationship between BW loss and occurrence of BRONJ, between PD type (localized or generalized) and oral lesion location (maxilla or mandible), and between PD type (localized or generalized) and total number of BRONJ cases. Multiple logistic regression analysis was used to determine whether dose and duration were significant predictors of BRONJ prevalence among individual dose levels, and when the doses were stratified in three groups: control (ZOL 0), OP (ZOL 8) and oncology doses (ZOL 20, 50, 125). Data are expressed as Mean ± SD. For ABH, osteocyte lacunae, and ELISA, one‐way ANOVA with Holm‐Sidak post hoc analysis was used to assess intergroup differences. When assumptions of data normality were not met, a Kruskal–Wallis ANOVA on Ranks followed by Dunn's multiple comparison was applied. Spearman rank correlation was used to determine whether PD Score increased with age. Student's *t* test was used to determine differences in the alveolar ridge width measurements between ZOL 0 and ZOL‐treated rats. Two‐way ANOVA was used to assess the main effects of ZOL dose and duration on pQCT measurements. Two‐way ANOVA was also used to determine the main effects of oral health status (healthy, PD, BRONJ) and ZOL dose on TRAP+/B.Pm. The means of the ZOL dose groups were collapsed into a single PD group for comparison with BRONJ using Student's *t* test. A power calculation was performed using SAS PROC POWER logistic regression. This experiment has 80% power to determine significant differences in BRONJ prevalence between control and 125 µg/kg BW ZOL. In all cases, *p < *0.05 was considered statistically significant.

## 3 RESULTS

### 3.1 Periodontitis status (ZOL 0 rats)

All quadrants in baseline rats had *GQG* 0 with PD Score 0 ± 0. PD was prevalent in 60, 56, 80 and 92% of ZOL 0 rats at 12, 18, 24 and 30 weeks, respectively (Figure 2a). In rats with PD, PD Score ranged from 1–4, and there was a significant positive correlation between PD Score and duration on HSC diet (*R* = 0.496, *p = *0.00124) (Figure 2b). There was significantly greater alveolar bone loss at 24 and 30 weeks than at 12 and 18 weeks in mandibles, and at 12, 24 and 30 weeks than at baseline in maxillae (Figure 2c). Moderate PD in rice rats (PD Score 3) rats showed bacterial plaque, apical migration of junctional epithelium, alveolar bone resorption, increased ABH, gingival hyperplasia, inflammatory infiltrate, and PDL disruption **(**Figure 2d**)**.

### 3.2 Prevalence of BRONJ‐like lesions

**Figure 3:**
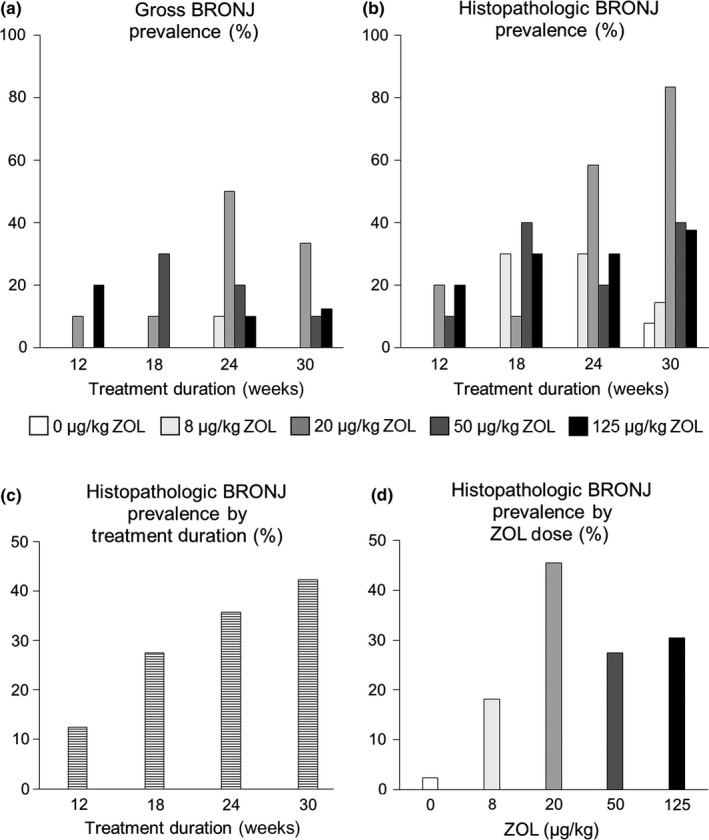
Prevalence of BRONJ. (a) Prevalence of gross BRONJ (high‐resolution photographs). One case was found at 24 weeks in the zoledronate (ZOL) OP dose; 23 cases were found after 12–30 weeks with ZOL oncology doses (20–125). (b) Prevalence of histopathologic BRONJ. One case was found in a ZOL 0 rat at 30 weeks. Eight cases were found at 18–30 weeks with the ZOL OP dose. Forty‐five cases were found at 12–30 weeks with ZOL oncology doses (20–125). Prevalence of histopathologic BRONJ cases within (c) each time point and (d) each dose group

The prevalence of gross BRONJ‐like lesions is shown (Figure 3a). Neither dose (*p* = 0.418) nor duration (*p* = 0.210) were significant predictors of gross BRONJ prevalence. When ZOL groups were stratified by ZOL 0, OP and oncologic doses (ZOL 20–125), gross BRONJ was prevalent in 0%, 2% (1/44) and 18% (23/130) of rats, respectively. Multiple logistic regression showed that dose (*p = *0.008), but not duration (*p = *0.165) was a significant predictor of gross BRONJ prevalence when stratified this way.

Histopathologic examination confirmed all 24 cases of gross BRONJ and revealed 30 additional cases, for a total of 54 cases of histopathologic BRONJ. The prevalence of histopathologic BRONJ is shown (Figure 3b**)**. Histopathologic BRONJ prevalence increased steadily with time throughout the experiment **(**Figure 3c), but histopathologic BRONJ prevalence reached a plateau at the ZOL 20 dose (Figure 3d). Multiple linear regression showed that duration (*p* < 0.001), but not dose (*p* = 0.076), was a significant predictor of overall histopathologic BRONJ prevalence. When ZOL groups were stratified by ZOL 0, OP and oncologic doses, histopathologic BRONJ was prevalent in 2% (1/42 rats), 18% (8/44 rats) and 35% (45/130 rats), respectively. Both ZOL dose (*p* < 0.001) and duration (*p* < 0.001) were significant predictors of histopathologic BRONJ prevalence when stratified this way. One maxilla met all criteria for BRONJ in a ZOL 0 rat at age 34 weeks.

### 3.3 Features of BRONJ lesions

#### 3.3.1 Gross and histopathologic

**Figure 4:**
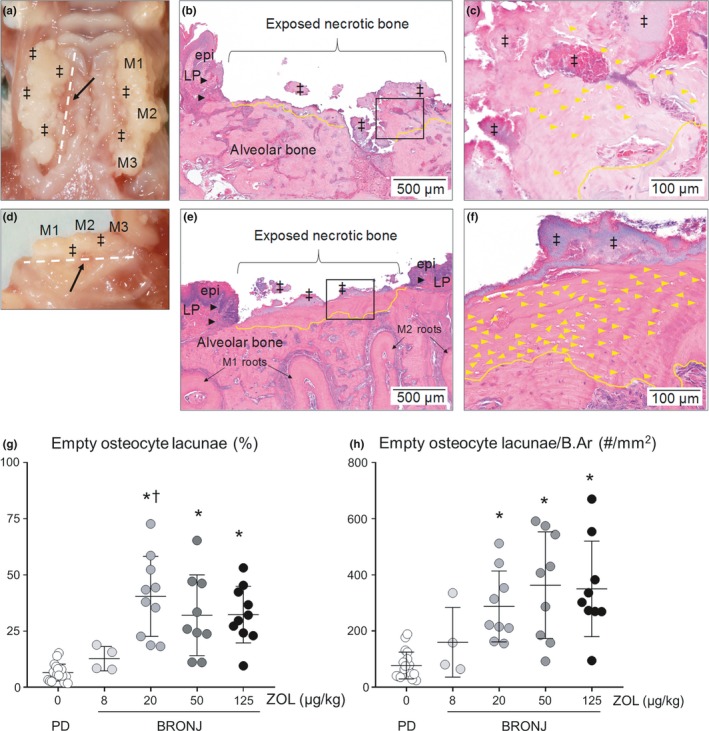
Features of necrotic bone in BRONJ‐affected maxillae and mandibles of rats injected with zoledronate (ZOL) at gross (a, d) and microscopic levels (b, c and e, f). High‐resolution photographs of gross BRONJ lesions in (a) maxillae and (d) mandible; note heavy plaque accumulation (‡) and ulcerated mucosa with exposed bone (arrow). White lines indicate approximate location of sections shown in b, c and e, f. Parasagittal sections through (b, c) the palatal bone of maxillae and (e, f) the mandibular lingual plate with H&E stain; ulcerated mucosa with inflammation (black arrowheads) in the lamina propria (LP) and epithelial (epi) hyperplasia; exposed bone surface is colonized by bacteria (‡). Border between healthy and necrotic bone is demarcated in yellow. (c, f) Higher magnification of exposed bone tissue shows extensive field of empty osteocyte lacunae (yellow arrowheads). (g) The percentage of empty osteocyte lacunae in alveolar bone was higher in BRONJ lesions in rats treated with ZOL 20, 50 or 125 compared to BRONJ lesions in ZOL 0 (*p < 0.05) and ZOL 8 (†*p* < 0.05) rats. (h) The number of empty osteocyte lacunae per bone area (mm^2^) was significantly higher in ZOL 20, 50 or 125 doses compared to ZOL 0 (*p < 0.05). Mean ± *SD*

Gross BRONJ‐like lesions were found in both maxillae (Figure 4a) and mandibles (Figure 4d) and showed exposed bone, ulcerated mucosa, plaque and irregular gingival contours around the margins of the molars. Gross oral lesions were distributed evenly between the maxillae (46%) and mandibles (54%) (*p* = 0.1953).

Histopathologic BRONJ‐like lesions in both maxillae (Figure 4b,c) and mandibles (Figure 4e,f) were characterized by confluent areas of empty osteocyte lacunae in bone exposed to the oral cavity, and the surface was usually colonized by bacteria. Mucosal and periodontal tissues around the margins of the lesions had marked inflammatory infiltrate. Haemorrhage, fibrosis, numerous reversal lines in bone and necrotic soft tissue were also apparent.

BRONJ‐like lesions occurred in different areas of alveolar bone, including interdental regions, the M1 interradicular bone, distal to M3, or on the hard palate or lingual plate. Necrotic bone was observed underneath intact mucosa in some sections. However, in all such cases, *exposed* necrotic bone was present in sections from adjacent levels within the same quadrant **(**Supporting Information Figure S3 **)**.

The percentage of empty osteocyte lacunae in BRONJ rats was significantly higher (*p* < 0.05) than in PD control rats in the oncologic ZOL dose groups (20–125) (Figure 4g), and similar results were found when empty osteocyte lacunae frequency was normalized to bone area (Figure 4h).

#### 3.3.2 MicroCT

**Figure 5:**
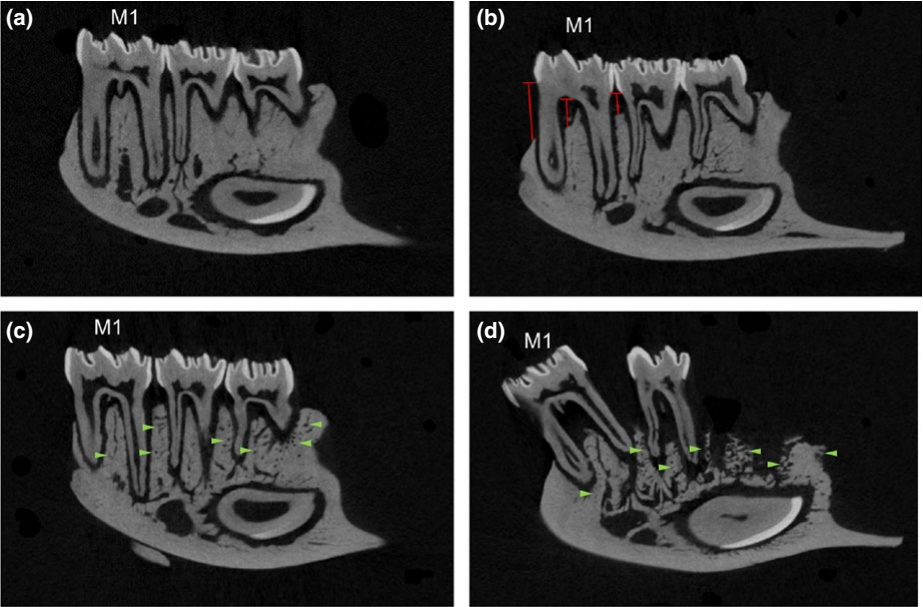
Representative 2D MicroCT slices of mandibles from rats treated with oncologic doses of zoledronate (ZOL) (20–125). (a) Normal mandible. (b) Mandibles with PD‐only showed loss of alveolar bone height mesial to M1, in the M1 furcation and in the M1M2 interdental region (red lines). (c) Mandibles with BRONJ showed mottled, honeycomb‐like appearance in the entire alveolar process (green arrowheads), but not the cortical bone of the inferior border of the mandible. (d) In more severe BRONJ, profound bone destruction, tooth migration and tooth loss were observed along with the honeycomb‐like appearance of the alveolar process

Periodontitis‐affected mandibles exhibited loss of ABH mesial to M1, in the M1 furcation, and in the M1M2 interdental region (Figure 5b), compared to normal mandibles (Figure 5a**)**. Mandibles affected by BRONJ‐like lesions exhibited large regions of mottled alveolar bone with numerous pores, giving the entire alveolar bone region a honeycomb‐like appearance (Figure 5c,d). In severe BRONJ‐like lesions, profound alveolar bone osteolysis, tooth migration, and spaces with apparent missing teeth were noted (Figure 5d). Histological analysis from quadrants shown in Figure 5c,d confirmed localized areas of exposed, necrotic bone at the M1M2 interdental alveolar bone crest and distal to M3.

#### 3.3.3 Alveolar bone osteoclasts

**Figure 6:**
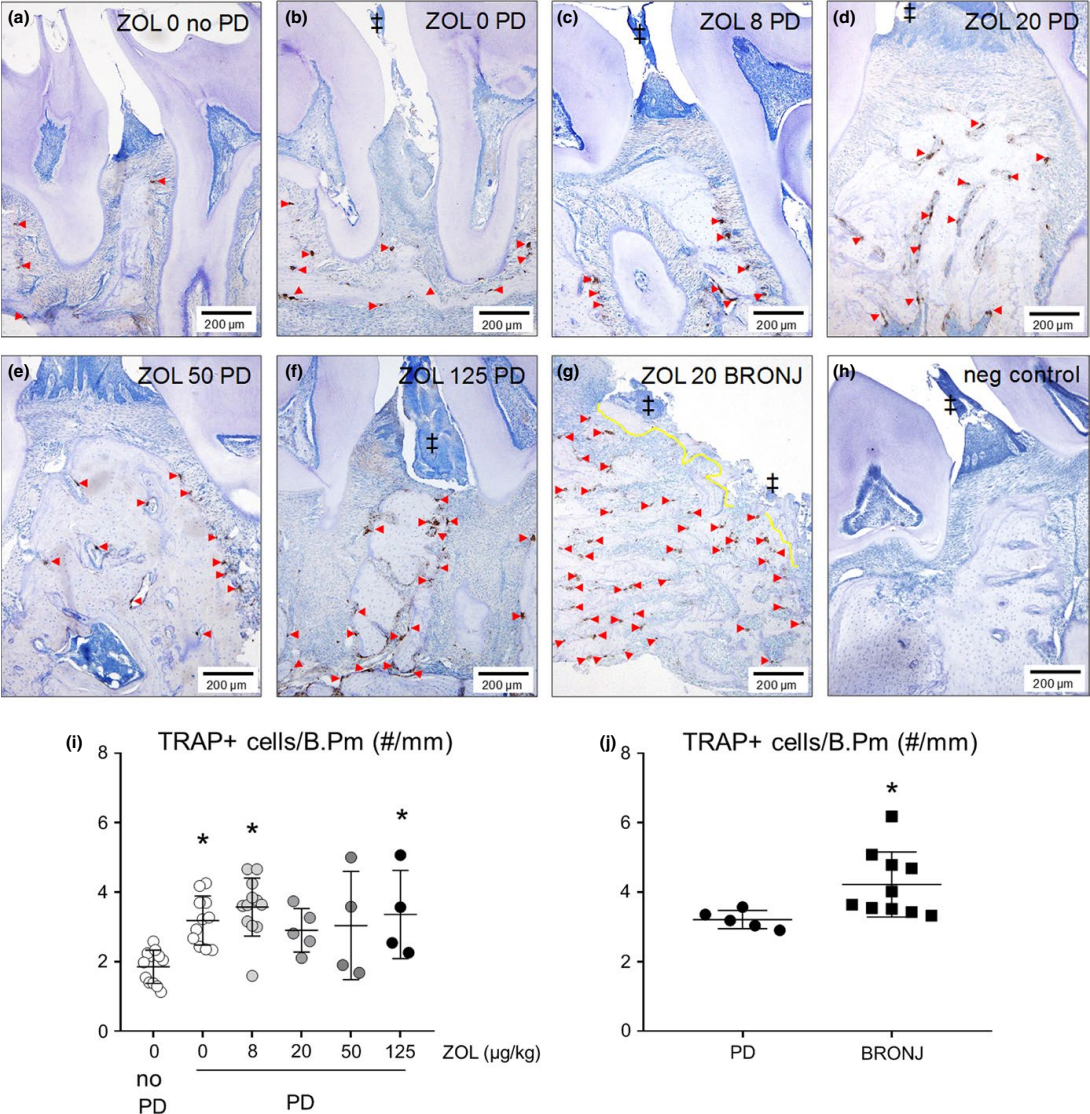
Osteoclasts in the maxillary alveolar bone of rice rats treated with zoledronate (ZOL). Representative images of TRAP+ immunolabelled osteoclasts (red arrow heads) on the maxillary alveolar bone surfaces of (a) rats with no PD treated with ZOL 0, and rats with PD treated with either (b) ZOL 0, (c) ZOL 8, (d) ZOL 20, (e) ZOL 50 or (f) ZOL 125. (g) Numerous TRAP+ osteoclasts in alveolar bone affected by BRONJ. Plaque (‡) is visible in PD‐affected maxillae and is directly attached to necrotic bone (demarcated in yellow) in BRONJ. (h) Absence of TRAP+ staining in negative control. (i) Number of TRAP+ osteoclasts per mm of bone perimeter (B.Pm) in alveolar bone of rats with no PD treated with ZOL 0, and rats with PD treated with various doses of ZOL (0–125); * indicates *p* < 0.05 compared to ZOL 0 no PD control. (j) TRAP+ cells per mm B.Pm in alveolar bone affected by PD or BRONJ. * indicates *p* < 0.05 compared to PD. Mean ± *SD*

Compared to ZOL 0 rats with no PD (Figure 6a), ZOL 0 rats with PD had more numerous visible multinucleated TRAP+ osteoclasts at maxillary alveolar bone surfaces (Figure 6b). TRAP+ cells were also visible on alveolar bone surfaces of ZOL‐treated rats with PD in all dose groups (Figure 6c–f), and numerous TRAP+ cells were visible on alveolar bone with BRONJ (Figure 6g**). **There was no TRAP+ staining detected in negative control sections (Figure 6h). Two‐way ANOVA revealed no significant effect of ZOL dose on TRAP+ cells/B.Pm (*p* = 0.827), but there was a significant effect of disease status (*p < *0.001), where alveolar bone with PD and BRONJ had significantly more TRAP+ cells/B.Pm, compared to the alveolar bone of rats with no PD treated with ZOL 0. There were more TRAP+ cells in maxillary alveolar bones in the ZOL 0 PD, ZOL 8 PD and ZOL 125 PD groups compared to the alveolar bones of ZOL 0 no PD rats (Figure 6i). Since there were no significant effects of ZOL dose, the means of each ZOL dose group were collapsed into a single PD group and compared to maxillary alveolar bone from rats with BRONJ. There were significantly higher numbers of TRAP+ cells/B.Pm (*p* = 0.037) in maxillae with BRONJ‐like lesions compared to maxillae with PD (Figure 6j).

#### 3.3.4 Skeletal effects of ZOL

**Figure 7:**
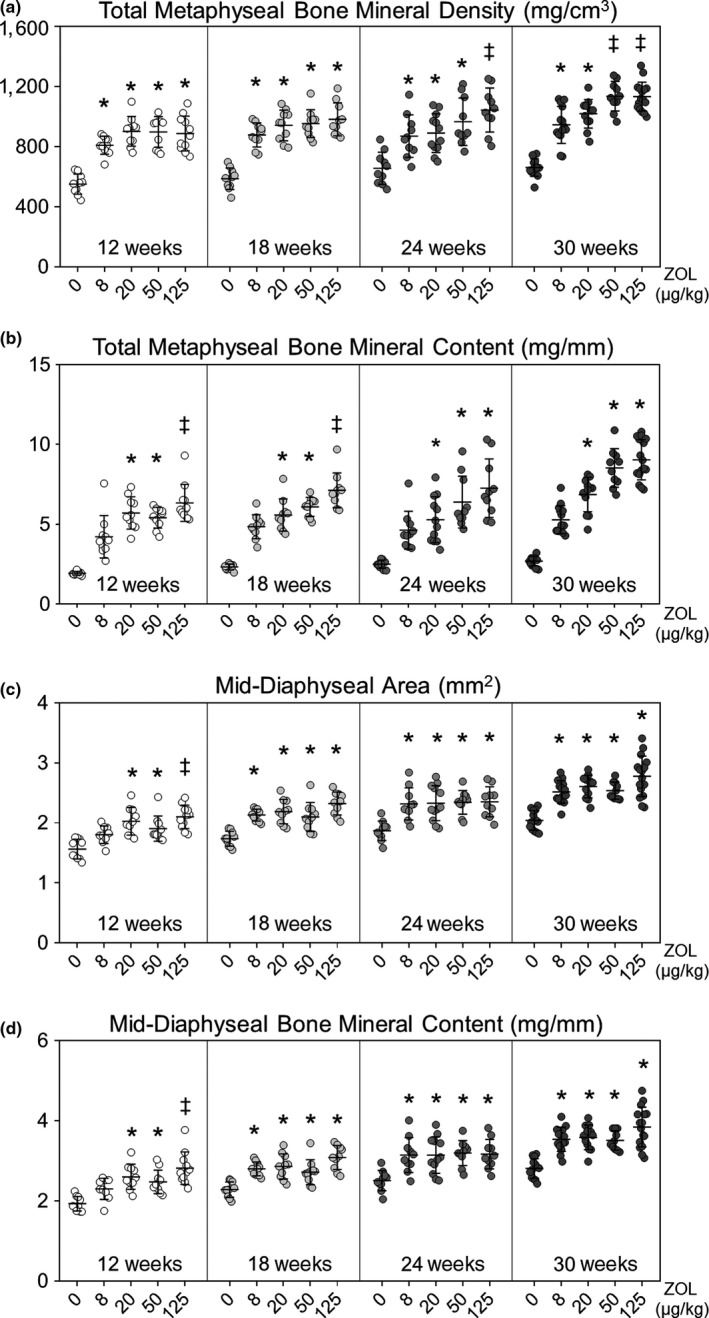
Peripheral quantitative computed tomography of the femoral distal metaphysis and mid‐diaphysis after treatment with zoledronate (ZOL) 0–125 for up to 30 weeks, starting at age 4 weeks. (a) Total distal metaphyseal volumetric bone mineral density (vBMD; mg/cm^3^); (b) total volumetric distal metaphyseal bone mineral content (vBMC; mg/mm); (c) total cortical bone area (mm^2^); and (d) cortical vBMC (mg/mm). The main effects of dose (*p* < 0.001) and time (*p* < 0.001) were significant for all measurements, and there were some significant differences among doses within each time point. However, there were no significant differences among the ZOL oncology doses at any time. *indicates *p < *0.05 compared to 0 ZOL; ‡ indicates *p < *0.05 compared to 0 ZOL and 8 ZOL. Mean ± *SD*

Peripheral quantitative computed tomography analysis confirmed the anti‐resorptive effects of ZOL in both the cancellous and cortical bone compartments. Total metaphyseal vBMD (Figure 7a) and vBMC (Figure 7b) were significantly higher in ZOL‐treated animals, and the response was dependent on both dose (*p* < 0.001) and duration (*p* < 0.001). The highest BMD was observed at 30 weeks in ZOL 50 and ZOL 125 groups, both of which were significantly different from the ZOL 0 and ZOL 8 groups. Both mid‐diaphyseal (cortical) area (Figure 7c) and mid‐diaphyseal vBMC (Figure 7d) were higher with dose (*p* < 0.001) and duration (*p* < 0.001). There were no significant differences among the oncologic doses (ZOL 20–125) at any time.

**Figure 8:**
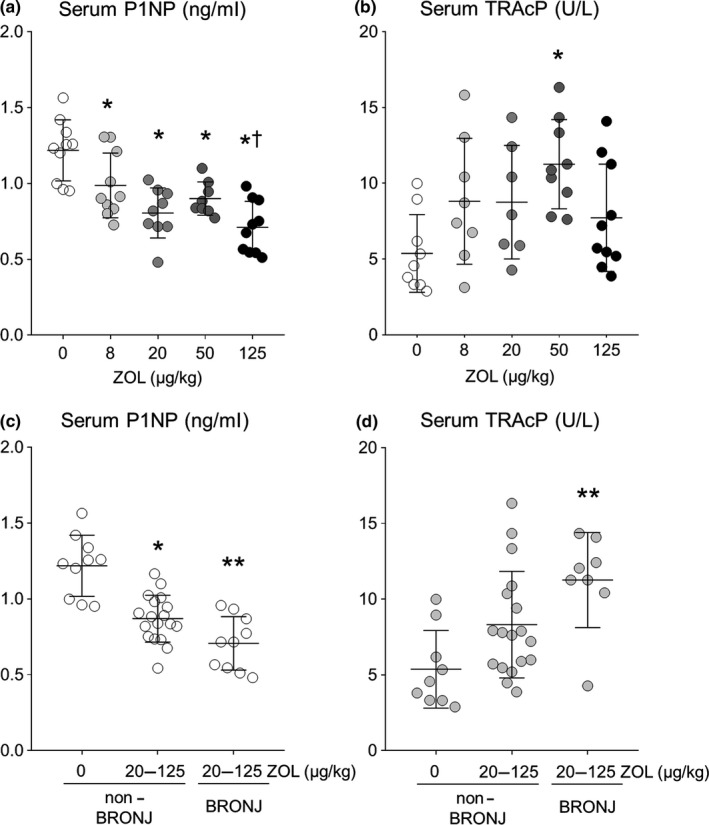
Serum bone turnover marker concentration in rice rats. (a) P1NP and (b) TRAcP 5b after treatment with ZOL 0–125 for 24 weeks. *p < 0.05 compared to ZOL 0; ^†^ *p* < 0.05 compared to ZOL 8. (c) P1NP and (d) TRAcP 5b in rats treated with ZOL 0 or oncologic ZOL doses (20–125) for 24 weeks and grouped by BRONJ status; *indicates *p* < 0.05 compared to non‐BRONJ ZOL 0; **indicates *p* < 0.05 compared to non‐BRONJ ZOL 0 and non‐BRONJ ZOL 20–125. Non‐BRONJ ZOL 0, *n* = 9; non‐BRONJ ZOL 20–125, *n* = 18; BRONJ ZOL 20–125, *n* = 8. Mean ± *SD*

Serum P1NP was dose‐dependently lower in ZOL‐treated rats (*p* < 0.001) (Figure 8a). All doses of ZOL resulted in significantly lower serum P1NP compared to ZOL 0 (*p* < 0.05). There were no differences in serum P1NP among the 20, 50 and 125 ZOL groups. Serum TRAcP 5b was significantly higher only at the ZOL 50 compared to other dose groups (Figure 8b). Serum P1NP was significantly lower in rats treated with ZOL 20–125 with BRONJ‐like lesions, compared to rats treated with either ZOL 0 or ZOL 20–125 without BRONJ (Figure 8c). Conversely, serum TRAcP 5b was significantly higher in rice rats treated with ZOL 20–125 with BRONJ‐like lesions compared to rice rats treated with either ZOL 0 or ZOL 20–125 without BRONJ (Figure 8d).

**Figure 9:**
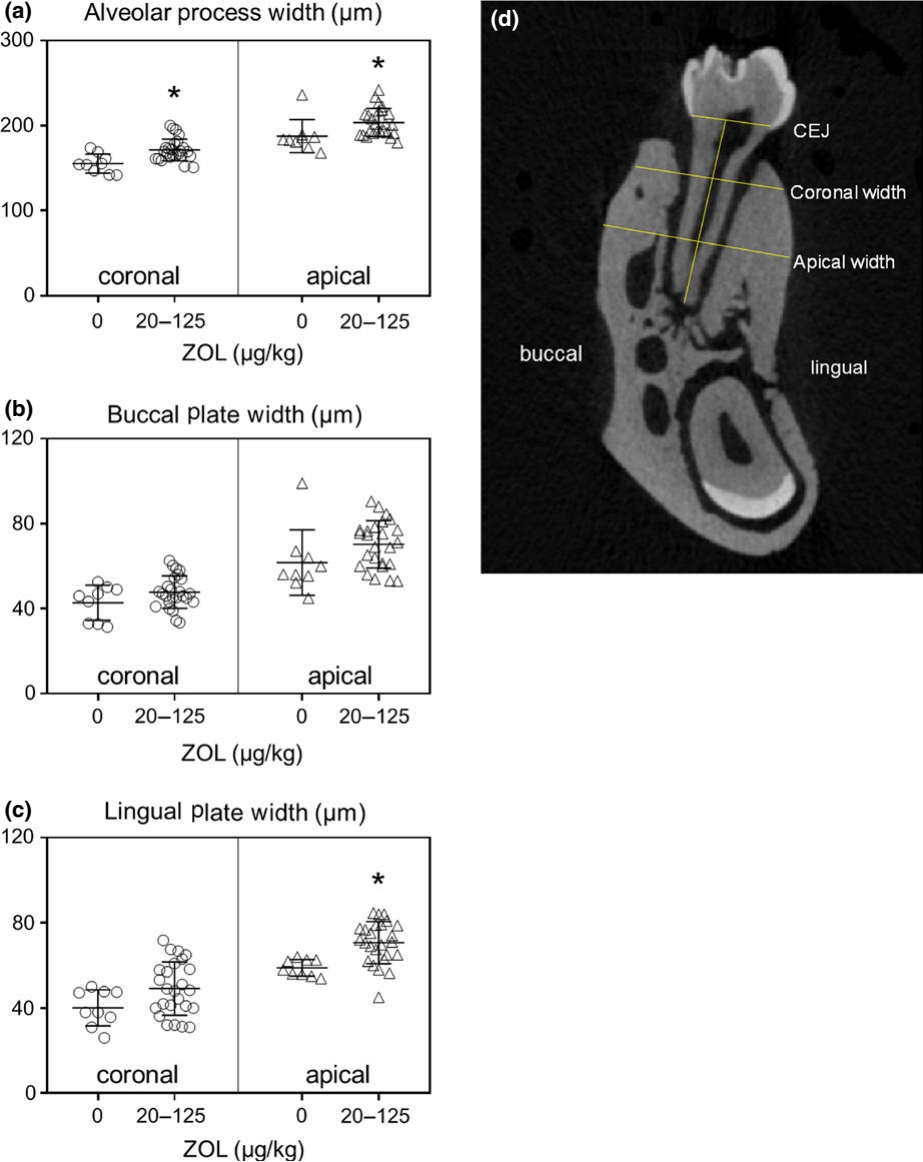
Width of alveolar process of mandibles in rice rats treated with ZOL 0 or oncologic ZOL doses (20–125). (a) total alveolar process width, (b) buccal plate width and (c) lingual plate width; (d) diagram of apical and coronal sites of measurement. *indicates *p < *0.05 compared to ZOL 0 at each measurement site. ZOL 0, *n* = 9; ZOL 20–125, *n* = 25. Mean ± *SD*

ZOL‐treated rats had significantly increased width of mandibular alveolar processes (*p* < 0.05) (Figure 9a). While buccal plate width of rats treated with ZOL 20–125 was the same as ZOL 0 control (Figure 9b), the lingual plate was significantly wider at the apical portion of the alveolar process (*p < *0.05) (Figure 9c).

## 4 DISCUSSION

This study demonstrates that longer duration of oncologic doses of ZOL is associated with a higher prevalence of BRONJ in rice rats that are simultaneously developing generalized PD. BRONJ prevalence appeared to plateau at an oncologic ZOL dose (ZOL 20) that was only 2.5‐fold higher than the OP dose. A similar plateau at the ZOL 20 dose was observed for the known anti‐resorptive effect of ZOL, as indicated by a plateau in bone mass. Finally, ZOL treatment and BRONJ‐like lesions produced tissue‐level effects on the jaws that were distinctive from both healthy rats, and rats with generalized PD that did not receive ZOL. The pattern of prevalence and the tissue‐level effects on the jaws support the overall hypothesis that systemic ZOL administration induces BRONJ‐like lesions in distinct patterns depending on the different types of local oral inflammatory conditions.

This study supports the role of local oral inflammation as a key component in the development of BRONJ that is not directly related to dental surgery. The current study, along with studies in other rodent models, has shown that BRONJ‐like lesions can arise from several types of oral inflammation processes or wounds, which is consistent with human MRONJ. BRONJ‐like lesions have been observed when ZOL treatment is combined with mechanically exposed dental pulp (Kang et al., 2013; Wayama et al., 2015), experimental PD via dental ligature (Li et al., 2016) and in association with localized intraoral lesions where impacted food and hair produce periodontal inflammation (Messer et al., 2018; de Molon et al., 2014). The requirement of local oral factors is also supported in the current study by a subpopulation of rice rats (32%) given oncologic doses of ZOL that never developed BRONJ despite receiving cumulative ZOL doses up to 1,000 µg/kg over 30 weeks. These rats had *GQG* of 0 (92%) or 1 (8%), and exhibited no BW loss at necropsy, suggesting healthy oral cavities. Similarly, Sprague Dawley rats treated up to 2 years with alendronate at up to 10 times the OP dose and ZOL at up to seven times the oncology dose show no BRONJ‐like lesions (Merck, 2018; Novartis, 2018), possibly because of the known resistance of rodents from the genus *Rattus* to develop PD (Graves, Kang, Andriankaja, Wada, & Rossa, 2012; Oz & Puleo, 2011; Struillou, Boutigny, Soueidan, & Layrolle, 2010; Weinberg & Bral, 1999).

While inflammation or infection from oral diseases, or direct compromise of the gingival barrier via a wound, appears to be crucial for developing BRONJ, evidence from rice rat studies suggests that the type of oral inflammation may produce BRONJ in distinctive patterns, and with distinctive features when bone resorption is suppressed by ZOL. In the FILP model, BRONJ‐like lesions developed primarily in maxillae (74%) compared to mandibles (26%) (*p* < 0.0001), and there was a significant increase in prevalence dependent on dose, but not duration of ZOL treatment (Messer et al., 2018). In the current study, rice rats fed the HSC diet developed generalized PD but not FILP. In this generalized PD model, BRONJ lesions were distributed evenly between maxillae and mandibles, and were associated with ZOL duration, but not dose. The FILP model also produced a significantly higher total prevalence of histopathologic BRONJ compared to the generalized PD model (*p < *0.0001), at 47% (82/174) vs 31% (53/171), respectively (Messer et al., 2018). The distinctive relationships between ZOL and BRONJ prevalence may reflect differences in the initiation and progression of the two types of PD. The highest BRONJ prevalence in the generalized PD model occurred at 30 weeks, while the highest prevalence of BRONJ in the FILP model occurred at 18 weeks and plateaued at later time points (Messer et al., 2018). This pattern is congruent with the time points in which the PD lesions become moderate to severe in each model, as FILP occurs in up to 75% of rice rats by age 16 weeks (Messer et al., 2018), while destruction of tissues in the generalized PD model was more prevalent and more severe in this study after 24–30 weeks. These data suggest that the FILP model may be more severe, and produces BRONJ‐like lesions more rapidly compared to generalized PD. The direct trauma to the soft tissues from the impacted materials of the FILP lesion may directly introduce microorganisms to periodontal tissues through the wound, which is in contrast to generalized PD, which is characterized by accumulation of plaque on tooth surfaces, and inflammation in the gingival and periodontal tissue. The different effects of the local oral factors on the time‐to‐onset of BRONJ in the two rice rat PD models may partly explain findings in humans, in which the time‐to‐onset of BRONJ after exposure to pARs is widely variable, ranging from 0.1 to 19.9 years (Fung et al., 2017).

The histopathological examination of BRONJ‐like lesions at the tissue level was consistent with previous studies in rice rats, where exposed necrotic bone with attached bacterial colonies was observed (Aguirre, Akhter, Kimmel, Pingel, Xia et al., 2012). In the current study, osteonecrosis was not found under intact mucosa in any of the 198 examined quadrants. Additionally, four lesions showed bone that was exposed, but vital. Taken together, these novel findings suggest that bone in the periodontal lesion of pAR‐treated subjects may become exposed before becoming necrotic. “Stage 0” MRONJ in humans, in which nonspecific clinical findings, radiographic changes and symptoms are reported without clinical evidence of necrotic bone, could have exposed, necrotic bone in deep periodontal pockets, or in periapical regions of severely carious or endodontically treated teeth with chronic infection. Patients taking pARs with clinical findings of moderate/advanced PD or periapical pathology, such as bleeding on probing or intermittent tooth sensitivity, may actually have exposed, necrotic bone in periodontal pockets or in the periapical region that is only histologically detectable.

One maxilla met all criteria for a BRONJ‐like lesion in a ZOL 0 rat at age 34 weeks, indicating that dead alveolar bone in rice rats with severe PD that were not treated with a pAR, can occasionally accumulate in sufficient quantity to be diagnosed as BRONJ. Bone necrosis is not generally observed in clinical examination of humans with severe PD who do not take pARs. This may be due to lack of routine tissue biopsy of severe PD lesions in humans, or because necrotic bone may be promptly removed by normal osteoclastic activity in the absence of pARs. Indeed, delayed removal of necrotic bone in humans taking pARs has been observed clinically in the treatment of femoral head osteonecrosis (Agarwala & Shah, 2011; Hong, Luo, Lin, Zhong, & Shi, 2014; Lai et al., 2005; Luo, Lin, Zhong, Yan, & Wang, 2014), suggesting that pARs may also cause persistence of necrotic bone induced by severe PD in the oral cavity, which could eventually result in clinically detectable MRONJ. Alternatively, oral ulceration with bone sequestration (Almazrooa & Woo, 2009; Farah & Savage, 2003; Khan et al., 2015; Palla, Burian, Klecker, Fliefel, & Otto, 2016; Peters, Lovas, & Wysocki, 1993; Sonnier & Horning, 1997) occurs in humans not taking pARs and may have some relevance to this finding.

MicroCT images of mandibles affected with BRONJ‐like lesions revealed mottling of alveolar bone distant from the necrotic bone tissue region identified histologically. These pores may be the result of osteoclast activity far from localized sites where necrotic bone has accumulated. This finding suggests that the presence of exposed necrotic bone tissue may activate bone resorption throughout the entire alveolar process of the afflicted quadrant, bearing similarity to a regional acceleratory phenomenon (RAP) (Duncan, Frame, Frost, & Arnstein, 1969; Frost, ; Mueller, Schilling, Minne, & Ziegler, 1991; Verna, 2016). Indeed, the number of TRAP+ osteoclasts per mm bone perimeter was significantly higher in maxillary alveolar bone of BRONJ‐affected rats, compared to maxillary alveolar bone of rats affected by PD, suggesting that exposed necrotic bone with a more extensive area of soft tissue damage and inflammation promote a greater recruitment and activation of osteoclasts.

MicroCT analysis also revealed that ZOL monotherapy can increase the width of the alveolar process in rice rats. This provides further evidence that in rats at risk for BRONJ, the entire alveolar process, not just the interdental and interradicular alveolar bone, is altered. This finding may also have important implications for patients with complete or partial removable dentures. Expanding bone width under the dentures may increase the likelihood of mucosal tissue compression, and initiate pressure sores and mucosal epithelium necrosis that can lead to bone exposure and MRONJ. MRONJ has been documented in patients wearing removable complete or partial dentures (Bamias et al., 2005; Kyrgidis et al., 2008; Levin, Laviv, & Schwartz‐Arad, 2007; Niibe, Ouchi, Iwasaki, Nakagawa, & Horie, 2015; Tsuji, Watanabe, Nakayama, Goto, & Kurita, 2017; Zarychanski, Elphee, Walton, & Johnston, 2006). While observation of edentulous alveolar ridges in pAR patients with cone beam CT may be currently limited by the conventional 0.125 mm voxel size, these findings suggest that paying close attention to the condition of the mucosa underlying removable dentures in pAR patients may reduce the incidence of MRONJ not related to dental surgery.

Oncologic doses of ZOL resulted in a roughly twofold higher prevalence of BRONJ compared to the OP dose (35% vs 18%, respectively), supporting the idea that oncologic doses of ZOL result in higher BRONJ prevalence. However, the prevalence of BRONJ was 38% (20/53) in rats treated with ZOL 20, which is only 2.5‐fold higher than the OP dose, and at the low end of the oncologic range, while the prevalence of BRONJ with ZOL 125, roughly 16‐fold higher than the OP dose, was only 26% (14/53). These data most likely indicate a prevalence plateau beginning at ZOL 20. A similar plateau was observed in bone mass and systemic measurement of bone formation marker P1NP. Specifically, ZOL increased femoral BMD and BMC, which plateaued around the ZOL 20 dose, and serum P1NP was reduced to similar levels by ZOL after 24 weeks of treatment. Serum TRAcP 5b was not significantly altered by ZOL except at the ZOL 50 dose, which was higher than control (ZOL 0) and other ZOL doses. Together, these findings suggest that BRONJ prevalence may be associated specifically with the well‐characterized anti‐resorptive effect of ZOL, and that the primary systemic effect of ZOL on osteoclasts in this model is likely attributed to decreased osteoclast activity, rather than decreased osteoclast numbers. When levels of serum turnover markers were stratified by BRONJ status, ZOL‐treated rats with BRONJ had significantly lower P1NP and significantly higher TRAcP 5b compared to ZOL‐treated rats without BRONJ. These findings indicate that BRONJ was associated with significantly suppressed systemic bone formation and high numbers of osteoclasts. Together, these findings have important implications in clinical studies that attempt to reduce the cumulative dose of pARs in oncology patients to reduce the incidence of MRONJ (Amadori et al., 2013; Corso et al., 2007; Himelstein et al., 2017; Hortobagyi et al., 2017). If both prevalence of MRONJ and prevention of skeletal‐related events are associated primarily with anti‐resorptive activity, then lowering the pAR dose enough to reduce MRONJ prevalence may coincidently compromise the efficacy of pARs in reducing skeletal‐related events.

The local effects of ZOL, PD and BRONJ on the number of osteoclasts present in maxillary alveolar bones of the experimental rice rats were also histopathologically evaluated. In this study, increased numbers of TRAP+ osteoclasts and increased loss of ABH were observed in ZOL 0 rats with PD, compared to ZOL 0 healthy rats, which represent well‐established pathologic features of PD. The addition of ZOL treatment to rats with PD did not result in any significant alteration in the number of TRAP+ cells at any dose. These findings suggest that: 1) the inflammation associated with PD plays an important role in the increased number of osteoclasts observed in maxillary alveolar bones of rice rats, but 2) ZOL does not have any significant influence in affecting the number of osteoclasts in the jaws, even at very high oncologic doses. This is in contrast with the findings published by Weinstein et al. of human transilial biopsy samples from osteoporosis patients treated with alendronate, which showed higher numbers of osteoclasts in cancellous bone of patients treated with an osteoporosis dose of 10 mg/day compared to placebo, but no effects in osteoclast number at 1 or 5 mg/day (Weinstein, Roberson, & Manolagas, 2009). These discrepant findings at the bone tissue level between osteoporosis patients and rice rats may be due to variation in the dose (osteoporosis vs oncologic) and type of bisphosphonate (ZOL vs alendronate), and in particular, differences in the specific bone type and microenvironments associated with PD and osteoporosis. Maxillary alveolar bone is anatomically and functionally different from the trabecular bone from the pelvis. In addition, jawbones of PD‐affected rats are in close proximity to pathogenic microbes of the oral cavity. The inflammatory microenvironment of PD therefore bears limited similarity to the non‐inflamed microenvironment in trabecular bone of the iliac crest. Taken together, these findings support the idea that the unique anatomical and physiological features of alveolar bone play an important role in the specificity of osteonecrosis to the jaws.

The design of this study limits the interpretation of our findings in a few important ways. First, ZOL injections began in normal, periodontally healthy rice rats. Two injections occurred at ages 4 and 8 weeks, when the skeleton is considered juvenile. Since MRONJ is an adverse event that occurs in humans first exposed to pARs in adulthood, future rice rat studies should begin administration of MRONJ‐inducing medications after the skeleton has matured (after age 12 weeks) to improve the parallel with the human condition. Second, human MRONJ also occurs in the setting of cancer or osteoporosis where patients, unlike rice rats, not only have a primary disease, but also receive other types of medications. These underlying health issues, or drug treatments, particularly in cancer patients, may elevate the risk of MRONJ. For example, corticosteroids, which negatively influence healing (Rubin & Palestine, 1989; Zitelli, 1987), are frequently used in treatment protocols for patients with cancer or autoimmune/inflammatory diseases that cause secondary osteoporosis (e.g., rheumatoid arthritis), and may suppress healing of oral wounds. This could account for the observation that humans exposed to oncology doses of ZOL have a roughly 200‐fold higher prevalence of MRONJ than osteoporosis patients (Coleman, Marshall et al., 2011; Coleman, Woodward et al., 2011; Grbic et al., 2010; Rathbone et al., 2013; Ruggiero et al., 2014), which is much higher compared to rice rat data, where oncology doses produced only 2‐ to 10‐fold higher BRONJ prevalence compared to the OP dose after 30 weeks. Third, this study is limited by our inability to fully examine PD and BRONJ in live animals. Thirty‐six per cent of BRONJ cases were found in rice rats with no gross oral cavity lesions (*GQG* < 1), but were symptomatic in the sense that the rats experienced loss of body weight. Diagnostic criteria available in the clinical setting (e.g., periodontal probing, patient‐reported pain, dental radiography, cone beam CT) cannot be achieved in a rodent study, so the exact progression of PD and BRONJ, or any spontaneous healing, could not be assessed. Finally, observations of BRONJ in rice rats at necropsy did not allow assessment of the duration of bone exposure. Longitudinal observations and oral examinations in live rice rats in future studies may address some of these limitations.

The reduction in morbidities and mortality, along with the low risk of MRONJ, strongly favours the continued use of pARs in humans. Therefore, completely describing the aetiology and pathophysiology of MRONJ is more important than ever to patient safety, and could provide evidence that allows development of prevention and treatment strategies for both dental surgery‐related and non‐surgery‐related MRONJ. Additionally, recent case reports describe patients with MRONJ who were not exposed to anti‐resorptive or anti‐angiogenic medications (Aghaloo & Tetradis, 2017), suggesting that MRONJ remains an emerging disease that may be associated with yet other medical conditions or drugs.

## 5 CONCLUSION

This study demonstrates a duration‐dependent increase in the prevalence of BRONJ with oncologic ZOL in rice rats with progressive generalized PD. BRONJ prevalence plateaued at a dose only 2.5‐fold higher than prevalence with the osteoporosis dose and was consistent with a similar plateau in the anti‐resorptive effects of ZOL. BRONJ and ZOL also produced unique alterations at the tissue level compared to PD and healthy rats treated with ZOL. The findings demonstrate that local oral risk factors play a key role in establishing an environment in which systemic medications can induce BRONJ, but that different local factors may interact with pARs in distinctive ways.

## CONFLICT OF INTEREST

The authors have no conflicts of interest. CVP's Institution receives research support from Bayer. CVP receives a small amount of salary support from the research support and royalties from UpToDate for writing for Bayer.

## AUTHOR CONTRIBUTIONS

Study design: JIA, DBK. Study conduct: JGM, JIA, JLMC, JMJ, EJC; Data collection: JGM, JIA, DBK, JLMC, JMJ, EJC, RI, EGP, JFY. Data analysis: JGM, JIA, DBK. Data interpretation: JGM, JIA, DBK, LK, CVP, JFY. Drafting manuscript: JGM, JIA, DBK. Revising manuscript content: JGM, JIA, DBK, JLMC, JMJ, EJC, CVP, LK, JFY, CVP. Approving final version of manuscript: JGM, JIA, DBK, JLMC, JMJ, EJC, RI, CVP, LK, EGP, JFY, CVP. JGM and JIA take responsibility for the integrity of the data analysis.

## References

[odi13052-bib-0001] Agarwala, S. , & Shah, S. B. (2011). Ten‐year follow‐up of avascular necrosis of femoral head treated with alendronate for 3 years. Journal of Arthroplasty, 26, 1128–1134. 10.1016/j.arth.2010.11.010 21256699

[odi13052-bib-0002] Aghaloo, T. , Hazboun, R. , & Tetradis, S. (2015). Pathophysiology of osteonecrosis of the jaws. Oral and Maxillofacial Surgery Clinics of North America, 27, 489–496. 10.1016/j.coms.2015.06.001 26412796 PMC4908822

[odi13052-bib-0003] Aghaloo, T. L. , & Tetradis, S. (2017). Osteonecrosis of the jaw in the absence of antiresorptive or antiangiogenic exposure: a series of 6 cases. Journal of Oral and Maxillofacial Surgery, 75, 129–142. 10.1016/j.joms.2016.07.019 27569557 PMC5527276

[odi13052-bib-0004] Aghaloo, T. L. , Cheong, S. , Bezouglaia, O. , Kostenuik, P. , Atti, E. , Dry, S. M. , … Tetradis, S. (2014). RANKL inhibitors induce osteonecrosis of the jaw in mice with periapical disease. Journal of Bone and Mineral Research, 29, 843–854. 10.1002/jbmr.2097 24115073 PMC4476544

[odi13052-bib-0005] Aghaloo, T. L. , Kang, B. , Sung, E. C. , Shoff, M. , Ronconi, M. , Gotcher, J. E. , … Tetradis, S. (2011). Periodontal disease and bisphosphonates induce osteonecrosis of the jaws in the rat. Journal of Bone and Mineral Research, 26, 1871–1882. 10.1002/jbmr.379 21351151 PMC3596511

[odi13052-bib-0006] Aguirre, J. I. , Akhter, M. , Kimmel, D. , Pingel, J. , Xia, X. , Williams, A. , … Wronski, T. J. (2012). Enhanced alveolar bone loss in a model of non‐invasive periodontitis in rice rats. Oral Diseases, 18, 459–468. 10.1111/j.1601-0825.2011.01893.x 22233442 PMC3326220

[odi13052-bib-0007] Aguirre, J. I. , Akhter, M. P. , Kimmel, D. B. , Pingel, J. E. , Williams, A. , Jorgensen, M. , … Wronski, T. J. (2012). Oncologic doses of zoledronic acid induce osteonecrosis of the jaw‐like lesions in rice rats (*Oryzomys palustris*) with periodontitis. Journal of Bone and Mineral Research, 27, 2130–2143. 22623376 10.1002/jbmr.1669 PMC3436957

[odi13052-bib-0008] Aguirre, J. I. , Akhter, M. P. , Neuville, K. G. , Trcalek, C. R. , Leeper, A. M. , Williams, A. A. , … Kimmel, D. B. (2016). Age‐related periodontitis and alveolar bone loss in rice rats. Archives of Oral Biology, 73, 193–205. 10.1016/j.archoralbio.2016.10.018 27771588 PMC5539967

[odi13052-bib-0009] Aguirre, J. I. , Edmonds, K. , Zamora, B. , Pingel, J. , Thomas, L. , Cancel, D. , … Wronski, T. J. (2015). Breeding, husbandry, veterinary care, and hematology of marsh rice rats (*Oryzomys palustris*), a small animal model for periodontitis. Journal of the American Association for Laboratory Animal Science, 54, 51–58. 25651091 PMC4311742

[odi13052-bib-0010] Aljohani, S. , Fliefel, R. , Ihbe, J. , Kuhnisch, J. , Ehrenfeld, M. , & Otto, S. (2017). What is the effect of anti‐resorptive drugs (ARDs) on the development of medication‐related osteonecrosis of the jaw (MRONJ) in osteoporosis patients: A systematic review. Journal of Cranio‐Maxillo‐Facial Surgery, 45, 1493–1502. 10.1016/j.jcms.2017.05.028 28687467

[odi13052-bib-0011] Almazrooa, S. A. , & Woo, S. B. (2009). Bisphosphonate and nonbisphosphonate‐associated osteonecrosis of the jaw: A review. Journal of the American Dental Association, 140, 864–875. 19571050 10.14219/jada.archive.2009.0280

[odi13052-bib-0012] Amadori, D. , Aglietta, M. , Alessi, B. , Gianni, L. , Ibrahim, T. , Farina, G. , … Ripamonti, C. I. (2013). Efficacy and safety of 12‐weekly versus 4‐weekly zoledronic acid for prolonged treatment of patients with bone metastases from breast cancer (ZOOM): A phase 3, open‐label, randomised, non‐inferiority trial. The Lancet Oncology, 14, 663–670. 10.1016/S1470-2045(13)70174-8 23684411

[odi13052-bib-0013] Auskaps, A. , Gupta, O. , & Shaw, J. (1957). Periodontal disease in the rice rat. III. Survey of dietary influences. Journal of Nutrition, 63, 325–343. 10.1093/jn/63.3.325 13481772

[odi13052-bib-0014] Bamias, A. , Kastritis, E. , Bamia, C. , Moulopoulos, L. A. , Melakopoulos, I. , Bozas, G. , … Dimopoulos, M. A. (2005). Osteonecrosis of the jaw in cancer after treatment with bisphosphonates: Incidence and risk factors. Journal of Clinical Oncology, 23, 8580–8587. 10.1200/jco.2005.02.8670 16314620

[odi13052-bib-0015] Beck, D. T. , Yarrow, J. F. , Beggs, L. A. , Otzel, D. M. , Ye, F. , Conover, C. F. , … Borst, S. E. (2014). Influence of aromatase inhibition on the bone‐protective effects of testosterone. Journal of Bone and Mineral Research, 29, 2405–2413. 24764121 10.1002/jbmr.2265 PMC8366408

[odi13052-bib-0016] Bouxsein, M. , Boyd, S. K. , Christiansen, B. A. , Guldberg, R. , Jepsen, K. J. , & Muller, R. (2010). Guidelines for assessment of bone microstructure in rodents using micro‐computed tomography. Journal of Bone and Mineral Research, 25, 1468–1486. 20533309 10.1002/jbmr.141

[odi13052-bib-0017] Carlson, E. R. , & Schlott, B. J. (2014). Anti‐resorptive osteonecrosis of the jaws: Facts forgotten, questions answered, lessons learned. Oral and Maxillofacial Surgery Clinics of North America, 26, 171–191. 10.1016/j.coms.2014.01.005 24630868

[odi13052-bib-0018] Coleman, R. E. , Marshall, H. , Cameron, D. , Dodwell, D. , Burkinshaw, R. , Keane, M. , … Bell, R. (2011). Breast‐cancer adjuvant therapy with zoledronic acid. New England Journal of Medicine, 365, 1396–1405. 21995387 10.1056/NEJMoa1105195

[odi13052-bib-0019] Coleman, R. , Woodward, E. , Brown, J. , Cameron, D. , Bell, R. , Dodwell, D. , … Thorpe, H. (2011). Safety of zoledronic acid and incidence of osteonecrosis of the jaw (ONJ) during adjuvant therapy in a randomised phase III trial (AZURE: BIG 01–04) for women with stage II/III breast cancer. Breast Cancer Research and Treatment, 127, 429–438. 10.1007/s10549-011-1429-y 21394500

[odi13052-bib-0020] Corso, A. , Varettoni, M. , Zappasodi, P. , Klersy, C. , Mangiacavalli, S. , Pica, G. , & Lazzarino, M. (2007). A different schedule of zoledronic acid can reduce the risk of the osteonecrosis of the jaw in patients with multiple myeloma. Leukemia, 21, 1545–1548. 17410188 10.1038/sj.leu.2404682

[odi13052-bib-0021] de Molon, R. S. , Cheong, S. , Bezouglaia, O. , Dry, S. M. , Pirih, F. , Cirelli, J. A. , … Tetradis, S. (2014). Spontaneous osteonecrosis of the jaws in the maxilla of mice on antiresorptive treatment: A novel ONJ mouse model. Bone, 68, 11–19. 10.1016/j.bone.2014.07.027 25093262 PMC4476062

[odi13052-bib-0022] Dimopoulos, M. A. , Kastritis, E. , Bamia, C. , Melakopoulos, I. , Gika, D. , Roussou, M. , … Bamias, A. (2009). Reduction of osteonecrosis of the jaw (ONJ) after implementation of preventive measures in patients with multiple myeloma treated with zoledronic acid. Annals of Oncology, 20, 117–120. 18689864 10.1093/annonc/mdn554

[odi13052-bib-0023] Du, H. , Gao, M. , Qi, C. , Liu, S. , & Lin, Y. (2010). Drug‐induced gingival hyperplasia and scaffolds: They may be valuable for horizontal food impaction. Medical Hypotheses, 74, 984–985. 10.1016/j.mehy.2010.01.013 20172659

[odi13052-bib-0024] Duncan, H. , Frame, B. , Frost, H. , & Arnstein, A. R. (1969). Regional migratory osteoporosis. Southern Medical Journal, 62, 41–44. 5766431 10.1097/00007611-196901000-00009

[odi13052-bib-0025] Eleutherakis‐Papaiakovou, E. , & Bamias, A. (2017). Antiresorptive treatment‐associated ONJ. European Journal of Cancer Care, 20, 8–24. 10.1111/ecc.12787 29063702

[odi13052-bib-0026] Farah, C. S. , & Savage, N. W. (2003). Oral ulceration with bone sequestration. Australian Dental Journal, 48, 61–64. 10.1111/j.1834-7819.2003.tb00011.x 14640160

[odi13052-bib-0027] Filleul, O. , Crompot, E. , & Saussez, S. (2010). Bisphosphonate‐induced osteonecrosis of the jaw: A review of 2,400 patient cases. Journal of Cancer Research and Clinical Oncology, 136, 1117–1124. 20508948 10.1007/s00432-010-0907-7 PMC11828172

[odi13052-bib-0028] Franco‐Pretto, E. , Pacheco, M. , Moreno, A. , Messa, O. , & Gnecco, J. (2014). Bisphosphonate‐induced osteonecrosis of the jaws: Clinical, imaging, and histopathology findings. Oral Surgery, Oral Medicine, Oral Pathology, and Oral Radiology, 118, 408–417. 10.1016/j.oooo.2014.04.017 25179128

[odi13052-bib-0029] Frost, H. M. (1983a). The regional acceleratory phenomenon: A review. Henry Ford Hospital Medical Journal, 31, 3–9. 6345475

[odi13052-bib-0030] Frost, H. M. (1983b). The regional acceleratory phenomenon. Orthopedic Clinics of North America, 1981, 725–726.

[odi13052-bib-0031] Frost, H. M. (2000a). The Frozen Shoulder syndrome plus other evidence and the Utah Paradigm suggest the syndrome's pathogenesis and new targets for collagenous tissue research. Journal of Musculoskeletal and Neuronal Interactions, 1, 113–119. 15758503

[odi13052-bib-0032] Frost, H. M. (2000b). The Utah paradigm of skeletal physiology: An overview of its insights for bone, cartilage and collagenous tissue organs. Journal of Bone and Mineral Metabolism, 18, 305–316. 11052462 10.1007/s007740070001

[odi13052-bib-0033] Fung, P. , Bedogni, G. , Bedogni, A. , Petrie, A. , Porter, S. , Campisi, G. , … Fedele, S. (2017). Time to onset of bisphosphonate‐related osteonecrosis of the jaws: A multicentre retrospective cohort study. Oral Diseases, 23, 477–483. 28039941 10.1111/odi.12632

[odi13052-bib-0034] Fusco, V. , Santini, D. , Armento, G. , Tonini, G. , & Campisi, G. (2016). Osteonecrosis of jaw beyond antiresorptive (bone‐targeted) agents: New horizons in oncology. Expert Opinion on Drug Safety, 15, 925–935. 10.1080/14740338.2016.1177021 27074901

[odi13052-bib-0035] Genco, R. J. , & Borgnakke, W. S. (2013). Risk factors for periodontal disease. Periodontology 2000, 62, 59–94. 23574464 10.1111/j.1600-0757.2012.00457.x

[odi13052-bib-0036] Gotcher, J. E. , & Jee, W. S. (1981). The progress of the periodontal syndrome in the rice rat. I. Morphometric and autoradiographic studies. Journal of Periodontal Research, 16, 275–291. 6458677 10.1111/j.1600-0765.1981.tb00976.x

[odi13052-bib-0037] Graves, D. T. , Kang, J. , Andriankaja, O. , Wada, K. , & Rossa, C. Jr (2012). Animal models to study host‐bacteria interactions involved in periodontitis. Frontiers of Oral Biology, 15, 117–132. 10.1159/000329675 22142960 PMC3766715

[odi13052-bib-0038] Grbic, J. T. , Black, D. M. , Lyles, K. W. , Reid, D. M. , Orwoll, E. , McClung, M. , … Su, G. (2010). The incidence of osteonecrosis of the jaw in patients receiving 5 milligrams of zoledronic acid: Data from the health outcomes and reduced incidence with zoledronic acid once yearly clinical trials program. Journal of the American Dental Association, 141, 1365–1370. 21037195 10.14219/jada.archive.2010.0082

[odi13052-bib-0039] Gupta, O. , & Shaw, J. (1956a). Periodontal disease in the rice rat. I. Anatomic and histopathologic findings. Oral Surgery, Oral Medicine, and Oral Pathology, 9, 592–603. 10.1016/0030-4220(56)90319-x 13322421

[odi13052-bib-0040] Gupta, O. , & Shaw, J. (1956b). Periodontal disease in the rice rat. II. Methods for the evaluation of the extent of periodontal disease. Oral Surgery, Oral Medicine, and Oral Pathology, 9, 727–735. 10.1016/0030-4220(56)90249-3 13349101

[odi13052-bib-0041] Hamadeh, I. S. , Ngwa, B. A. , & Gong, Y. (2015). Drug induced osteonecrosis of the jaw. Cancer Treatment Reviews, 41, 455–464. 25913713 10.1016/j.ctrv.2015.04.007

[odi13052-bib-0042] Hellstein, J. (2014). Osteochemonecrosis: An overview. Head & Neck Pathology, 8, 482–490. 10.1007/s12105-014-0583-z 25409847 PMC4245410

[odi13052-bib-0043] Himelstein, A. L. , Foster, J. C. , Khatcheressian, J. L. , Roberts, J. D. , Seisler, D. K. , Novotny, P. J. , … Shapiro, C. L. (2017). Effect of longer‐interval vs standard dosing of zoledronic acid on skeletal events in patients with bone metastases: A randomized clinical trial. JAMA, 317, 48–58. 28030702 10.1001/jama.2016.19425 PMC5321662

[odi13052-bib-0044] Hoefert, S. , & Eufinger, H. (2011). Relevance of a prolonged preoperative antibiotic regimen in the treatment of bisphosphonate‐related osteonecrosis of the jaw. Journal of Oral and Maxillofacial Surgery, 69, 362–380. 21122968 10.1016/j.joms.2010.06.200

[odi13052-bib-0045] Hong, Y. C. , Luo, R. B. , Lin, T. , Zhong, H. M. , & Shi, J. B. (2014). Efficacy of alendronate for preventing collapse of femoral head in adult patients with nontraumatic osteonecrosis. BioMed Research International, 2014, 716538 10.1155/2014/716538 25535614 PMC4244931

[odi13052-bib-0046] Hortobagyi, G. N. , Van, P. C. , Harker, W. G. , Gradishar, W. J. , Chew, H. , Dakhil, S. R. , … Lipton, A. (2017). Continued treatment effect of zoledronic acid dosing every 12 vs 4 weeks in women with breast cancer metastatic to bone: The OPTIMIZE‐2 randomized clinical trial. JAMA Oncology, 3, 906–912. 28125763 10.1001/jamaoncol.2016.6316 PMC5824238

[odi13052-bib-0047] Kang, B. , Cheong, S. , Chaichanasakul, T. , Bezouglaia, O. , Atti, E. , Dry, S. M. , … Tetradis, S. (2013). Periapical disease and bisphosphonates induce osteonecrosis of the jaws in mice. Journal of Bone and Mineral Research, 28, 1631–1640. 10.1002/jbmr.1894 23426919 PMC3688704

[odi13052-bib-0048] Ke, H. Z. , Qi, H. , Chidsey‐Frink, K. L. , Crawford, D. T. , & Thompson, D. D. (2001). Lasofoxifene (CP‐336,156) protects against the age‐related changes in bone mass, bone strength, and total serum cholesterol in intact aged male rats. Journal of Bone and Mineral Research, 16, 765–773. 10.1359/jbmr.2001.16.4.765 11316005

[odi13052-bib-0049] Khan, A. A. , Morrison, A. , Hanley, D. A. , Felsenberg, D. , McCauley, L. K. , O'Ryan, F. , … Compston, J. (2015). Diagnosis and management of osteonecrosis of the jaw: A systematic review and international consensus. Journal of Bone and Mineral Research, 30, 3–23. 25414052 10.1002/jbmr.2405

[odi13052-bib-0050] Khan, A. A. , Morrison, A. , Kendler, D. L. , Rizzoli, R. , Hanley, D. A. , Felsenberg, D. , … Compston, J. (2017). Case‐based review of osteonecrosis of the jaw (ONJ) and application of the international recommendations for management from the International Task Force on ONJ. Journal of Clinical Densitometry, 20, 8–24. 10.1016/j.jocd.2016.09.005 27956123

[odi13052-bib-0051] Kuroshima, S. , & Yamashita, J. (2013). Chemotherapeutic and antiresorptive combination therapy suppressed lymphangiogenesis and induced osteonecrosis of the jaw‐like lesions in mice. Bone, 56, 101–109. 23727433 10.1016/j.bone.2013.05.013

[odi13052-bib-0052] Kyrgidis, A. , Vahtsevanos, K. , Koloutsos, G. , Andreadis, C. , Boukovinas, I. , Teleioudis, Z. , … Triaridis, S. (2008). Bisphosphonate‐related osteonecrosis of the jaws: A case‐control study of risk factors in breast cancer patients. Journal of Clinical Oncology, 26, 4634–4638. 18574158 10.1200/JCO.2008.16.2768

[odi13052-bib-0053] Lai, K. A. , Shen, W. J. , Yang, C. Y. , Shao, C. J. , Hsu, J. T. , & Lin, R. M. (2005). The use of alendronate to prevent early collapse of the femoral head in patients with nontraumatic osteonecrosis. A randomized clinical study. Journal of Bone and Joint Surgery. American Volume, 87, 2155–2159. 10.2106/JBJS.D.02959 16203877

[odi13052-bib-0054] Levin, L. , Laviv, A. , & Schwartz‐Arad, D. (2007). Denture‐related osteonecrosis of the maxilla associated with oral bisphosphonate treatment. Journal of the American Dental Association, 138, 1218–1220. 17785387 10.14219/jada.archive.2007.0346

[odi13052-bib-0055] Li, C. L. , Lu, W. W. , Seneviratne, C. J. , Leung, W. K. , Zwahlen, R. A. , & Zheng, L. W. (2016). Role of periodontal disease in bisphosphonate‐related osteonecrosis of the jaws in ovariectomized rats. Clinical Oral Implants Research, 27, 1116–6. 10.1111/clr.12502 25371026

[odi13052-bib-0056] Luo, R. B. , Lin, T. , Zhong, H. M. , Yan, S. G. , & Wang, J. A. (2014). Evidence for using alendronate to treat adult avascular necrosis of the femoral head: A systematic review. Medical Science Monitor, 20, 2439–2447. 25424061 10.12659/MSM.891123 PMC4257480

[odi13052-bib-0057] Marx, R. E. (2003). Pamidronate (Aredia) and zoledronate (Zometa) induced avascular necrosis of the jaws: A growing epidemic. Journal of Oral and Maxillofacial Surgery, 61, 1115–1117. 12966493 10.1016/s0278-2391(03)00720-1

[odi13052-bib-0058] Matthews, D. C. , & Tabesh, M. (2004). Detection of localized tooth‐related factors that predispose to periodontal infections. Periodontology 2000, 34, 136–150. 14717860 10.1046/j.0906-6713.2003.003429.x

[odi13052-bib-0059] Merck (2018). Merck & Co., Inc, Internal Safety Assessment Department confidential document.

[odi13052-bib-0060] Messer, J. G. , Jiron, J. M. , Chen, H. Y. , Castillo, E. J. , Mendieta Calle, J. L. , Reinhard, M. K. , … Aguirre, J. I. (2017). Prevalence of food impaction‐induced periodontitis in conventionally housed marsh rice rats (*Oryzomys palustris*). Comparative Medicine, 67, 43–50. 28222838 PMC5310624

[odi13052-bib-0061] Messer, J. G. , Mendieta Calle, J. L. , Jiron, J. M. , Castillo, E. J. , Van Poznak, C. , Bhattacharyya, N. , … Aguirre, J. I. (2018). Zoledronic acid increases the prevalence of medication‐related osteonecrosis of the jaw in a dose dependent manner in rice rats (*Oryzomys palustris*) with localized periodontitis. Bone, 108, 79–88. 29289789 10.1016/j.bone.2017.12.025 PMC5828169

[odi13052-bib-0062] Montefusco, V. , Gay, F. , Spina, F. , Miceli, R. , Maniezzo, M. , Teresa, A. M. , … Corradini, P. (2008). Antibiotic prophylaxis before dental procedures may reduce the incidence of osteonecrosis of the jaw in patients with multiple myeloma treated with bisphosphonates. Leukaemia & Lymphoma, 49, 2156–2162. 10.1080/10428190802483778 19021059

[odi13052-bib-0063] Mueller, M. , Schilling, T. , Minne, H. W. , & Ziegler, R. (1991). A systemic acceleratory phenomenon (SAP) accompanies the regional acceleratory phenomenon (RAP) during healing of a bone defect in the rat. Journal of Bone and Mineral Research, 6, 401–410. 10.1002/jbmr.5650060412 1858523

[odi13052-bib-0064] Niibe, K. , Ouchi, T. , Iwasaki, R. , Nakagawa, T. , & Horie, N. (2015). Osteonecrosis of the jaw in patients with dental prostheses being treated with bisphosphonates or denosumab. Journal of Prosthodontic Research, 59, 3–5. 25212129 10.1016/j.jpor.2014.08.001

[odi13052-bib-0065] Novartis (2018). Novartis Pharmaceuticals, Internal Toxicology Department confidential document.

[odi13052-bib-0066] Nunn, M. E. (2003). Understanding the etiology of periodontitis: An overview of periodontal risk factors. Periodontology 2000, 32, 11–23. 12756030 10.1046/j.0906-6713.2002.03202.x

[odi13052-bib-0067] Offenbacher, S. (1996). Periodontal diseases: pathogenesis. Annals of Periodontology, 1, 821–878. 9118282 10.1902/annals.1996.1.1.821

[odi13052-bib-0068] Otto, S. , Schreyer, C. , Hafner, S. , Mast, G. , Ehrenfeld, M. , Sturzenbaum, S. , & Pautke, C. (2012). Bisphosphonate‐related osteonecrosis of the jaws ‐ characteristics, risk factors, clinical features, localization and impact on oncological treatment. Journal of Cranio‐Maxillo‐Facial Surgery, 40, 303–309. 10.1016/j.jcms.2011.05.003 21676622

[odi13052-bib-0069] Oz, H. S. , & Puleo, D. A. (2011). Animal models for periodontal disease. Journal of Biomedicine & Biotechnology, 2011, 1116–8. 10.1155/2011/754857 PMC3038839 21331345

[odi13052-bib-0070] Page, R. C. , Offenbacher, S. , Schroeder, H. E. , Seymour, G. J. , & Kornman, K. S. (1997). Advances in the pathogenesis of periodontitis: Summary of developments, clinical implications and future directions. Periodontology 2000, 14, 216–248. 9567973 10.1111/j.1600-0757.1997.tb00199.x

[odi13052-bib-0071] Palla, B. , Burian, E. , Klecker, J. R. , Fliefel, R. , & Otto, S. (2016). Systematic review of oral ulceration with bone sequestration. Journal of Cranio‐Maxillo‐Facial Surgery, 44, 257–264. 10.1016/j.jcms.2015.11.014 26782844

[odi13052-bib-0072] Peters, E. , Lovas, G. L. , & Wysocki, G. P. (1993). Lingual mandibular sequestration and ulceration. Oral Surgery, Oral Medicine, Oral Pathology, 75, 739–743. 10.1016/0030-4220(93)90433-5 8515988

[odi13052-bib-0073] Ramirez, L. , Lopez‐Pintor, R. M. , Casanas, E. , Arriba, L. , & Hernandez, G. (2015). New non‐bisphosphonate drugs that produce osteonecrosis of the jaws. Oral Health & Preventive Dentistry, 13, 385–393. 25884045 10.3290/j.ohpd.a34055

[odi13052-bib-0074] Rathbone, E. J. , Brown, J. E. , Marshall, H. C. , Collinson, M. , Liversedge, V. , Murden, G. A. , … Coleman, R. E. (2013). Osteonecrosis of the jaw and oral health‐related quality of life after adjuvant zoledronic acid: An adjuvant zoledronic acid to reduce recurrence trial subprotocol (BIG01/04). Journal of Clinical Oncology, 31, 2685–2691. 10.1200/JCO.2012.46.4792 23796998

[odi13052-bib-0075] Ripamonti, C. I. , Maniezzo, M. , Campa, T. , Fagnoni, E. , Brunelli, C. , Saibene, G. , … Cislaghi, E. (2009). Decreased occurrence of osteonecrosis of the jaw after implementation of dental preventive measures in solid tumour patients with bone metastases treated with bisphosphonates. The experience of the National Cancer Institute of Milan. Annals of Oncology, 20, 137–145. 10.1093/annonc/mdn526 18647964

[odi13052-bib-0076] Rubin, B. , & Palestine, A. G. (1989). Complications of corticosteroid and immunosuppressive drugs. International Ophthalmology Clinics, 29, 159–171. 2526794 10.1097/00004397-198902930-00006

[odi13052-bib-0077] Ruggiero, S. L. , Dodson, T. B. , Fantasia, J. , Goodday, R. , Aghaloo, T. , Mehrotra, B. , & O'Ryan, F. (2014). American Association of Oral and Maxillofacial Surgeons position paper on medication‐related osteonecrosis of the jaw–2014 update. Journal of Oral and Maxillofacial Surgery, 72, 1938–1956. 10.1016/j.joms.2014.04.031 25234529

[odi13052-bib-0078] Ryder, M. I. (1980). Histological and ultrastructural characteristics of the periodontal syndrome in the rice rat. I. General light microscopic observations and ultrastructural observations of initial inflammatory changes. Journal of Periodontal Research, 15, 502–515. 6449583 10.1111/j.1600-0765.1980.tb00308.x

[odi13052-bib-0079] Salvi, G. E. , Lawrence, H. P. , Offenbacher, S. , & Beck, J. D. (1997). Influence of risk factors on the pathogenesis of periodontitis. Periodontology 2000, 14 , 173–201. 10.1111/j.1600-0757.1997.tb00197.x 9567971

[odi13052-bib-0080] Song, M. , Alshaikh, A. , Kim, T. , Kim, S. , Dang, M. , Mehrazarin, S. , … Kim, R. H. (2016). Preexisting periapical inflammatory condition exacerbates tooth extraction‐induced bisphosphonate‐related osteonecrosis of the jaw lesions in mice. Journal of Endodontics, 42, 1641–1646. 10.1016/j.joen.2016.07.020 27637460 PMC5085836

[odi13052-bib-0081] Sonnier, K. E. , & Horning, G. M. (1997). Spontaneous bony exposure: A report of 4 cases of idiopathic exposure and sequestration of alveolar bone. Journal of Periodontology, 68, 758–762. 9287067 10.1902/jop.1997.68.8.758

[odi13052-bib-0082] Stopeck, A. T. , Lipton, A. , Body, J. J. , Steger, G. G. , Tonkin, K. , de Boer, R. H. , … Braun, A. (2010). Denosumab compared with zoledronic acid for the treatment of bone metastases in patients with advanced breast cancer: A randomized, double‐blind study. Journal of Clinical Oncology, 28, 5132–5139. 10.1200/JCO.2010.29.7101 21060033

[odi13052-bib-0083] Struillou, X. , Boutigny, H. , Soueidan, A. , & Layrolle, P. (2010). Experimental animal models in periodontology: A review. Open Dentistry Journal, 4, 37–47. 20556202 10.2174/1874210601004010037 PMC2885595

[odi13052-bib-0084] Tsuji, C. , Watanabe, H. , Nakayama, H. , Goto, M. , & Kurita, K. (2017). A case of bisphosphonate‐related osteonecrosis of the jaw in a patient with subpontic osseous hyperplasia. Case Reports in Dentistry, 2017, 9659761 10.1155/2017/9659761 28286679 PMC5329666

[odi13052-bib-0085] Ullman‐Cullere, M. H. , & Foltz, C. J. (1999). Body condition scoring: A rapid and accurate method for assessing health status in mice. Laboratory Animal Science, 49, 319–323. 10403450

[odi13052-bib-0086] Van den Wyngaert, T. , Wouters, K. , Huizing, M. T. , & Vermorken, J. B. (2011). RANK ligand inhibition in bone metastatic cancer and risk of osteonecrosis of the jaw (ONJ): Non bis in idem? *Support* . Care Cancer, 19, 2035–2040. 10.1007/s00520-010-1061-0 21203781

[odi13052-bib-0087] Vandone, A. M. , Donadio, M. , Mozzati, M. , Ardine, M. , Polimeni, M. A. , Beatrice, S. , … Scoletta, M. (2012). Impact of dental care in the prevention of bisphosphonate‐associated osteonecrosis of the jaw: A single‐center clinical experience. Annals of Oncology, 23, 193–200. 21427065 10.1093/annonc/mdr039

[odi13052-bib-0088] Verna, C. (2016). Regional acceleratory phenomenon. Frontiers of Oral Biology, 18, 28–35. 26599115 10.1159/000351897

[odi13052-bib-0089] Voss, P. J. , Poxleitner, P. , Schmelzeisen, R. , Stricker, A. , & Semper‐Hogg, W. (2017). Update MRONJ and perspectives of its treatment. Journal of Stomatology Oral and Maxillofacial Surgery, 118, 232–235. 10.1016/j.jormas.2017.06.012 28697987

[odi13052-bib-0090] Wayama, M. T. , Yoshimura, H. , Ohba, S. , Yoshida, H. , Matsuda, S. , Kobayashi, J. , … Sano, K. (2015). Diminished progression of periapical lesions with zoledronic acid in ovariectomized rats. Journal of Endodontics, 41, 2002–2007. 10.1016/j.joen.2015.08.029 26490005

[odi13052-bib-0091] Weinberg, M. A. , & Bral, M. (1999). Laboratory animal models in periodontology. Journal of Clinical Periodontology, 26, 335–340. 10382571 10.1034/j.1600-051x.1999.260601.x

[odi13052-bib-0092] Weinstein, R. S. , Roberson, P. K. , & Manolagas, S. C. (2009). Giant osteoclast formation and long‐term oral bisphosphonate therapy. New England Journal of Medicine, 360, 53–62. 19118304 10.1056/NEJMoa0802633 PMC2866022

[odi13052-bib-0093] Yamashita, J. , Koi, K. , Yang, D. Y. , & McCauley, L. K. (2011). Effect of zoledronate on oral wound healing in rats. Clinical Cancer Research, 17, 1405–1414. 21149614 10.1158/1078-0432.CCR-10-1614 PMC3060285

[odi13052-bib-0094] Yang, L. , Boyd, K. , Kaste, S. C. , Kamdem Kamdem, L. , Rahija, R. J. , & Relling, M. V. (2009). A mouse model for glucocorticoid‐induced osteonecrosis: Effect of a steroid holiday. Journal of Orthopaedic Research, 27, 169–175. 18683891 10.1002/jor.20733 PMC2718787

[odi13052-bib-0095] Yarrow, J. F. , Conover, C. F. , Beggs, L. A. , Beck, D. T. , Otzel, D. M. , Balaez, A. , … Borst, S. E. (2014). Testosterone dose dependently prevents bone and muscle loss in rodents after spinal cord injury. Journal of Neurotrauma, 31, 834–845. 10.1089/neu.2013.3155 24378197 PMC5911705

[odi13052-bib-0096] Yarrow, J. F. , Ye, F. , Balaez, A. , Mantione, J. M. , Otzel, D. M. , Chen, C. , … Vandenborne, K. (2014). Bone loss in a new rodent model combining spinal cord injury and cast immobilization. Journal of Musculoskeletal and Neuronal Interactions, 14, 255–266. 25198220 PMC8349504

[odi13052-bib-0097] Yoneda, T. , Hagino, H. , Sugimoto, T. , Ohta, H. , Takahashi, S. , Soen, S. , … Toyosawa, S. (2017). Antiresorptive agent‐related osteonecrosis of the jaw: Position Paper 2017 of the Japanese Allied Committee on Osteonecrosis of the Jaw. Journal of Bone and Mineral Metabolism, 35, 6–19. 10.1007/s00774-016-0810-7 28035494

[odi13052-bib-0098] Zarychanski, R. , Elphee, E. , Walton, P. , & Johnston, J. (2006). Osteonecrosis of the jaw associated with pamidronate therapy. American Journal of Hematology, 81, 73–75. 16369966 10.1002/ajh.20481

[odi13052-bib-0099] Zheng, L. Z. , Wang, J. L. , Kong, L. , Huang, L. , Tian, L. , Pang, Q. Q. , … Qin, L. (2018). Steroid‐associated osteonecrosis animal model in rats. Journal of Orthopaedic Translation, 13, 13–24. 10.1016/j.jot.2018.01.003 29662787 PMC5892381

[odi13052-bib-0100] Zitelli, J. (1987). Wound healing for the clinician. Advances in Dermatology, 2, 243–267. 3079257

