# Supplementary material for: Zoledronate treatment duration is linked to bisphosphonate‐related osteonecrosis of the jaw prevalence in rice rats with generalized periodontitis
Source: Oral Dis. 2019 Feb 19;25(4):1116–35. doi: 10.1111/odi.13052 (PMC6487955; doi:10.1111/odi.13052)
Supplement: Supplementary file 1 [file ODI-25-1116-s001.docx]

***Supporting Information 1. Sample sizes of necropsied rats for each zoledronate (ZOL) dose and duration subgroup and flow chart of selection criteria for histopathological examination of rats.***

Supplemental Table 1. Sample sizes for dose and duration groups for necropsied rats

Supplemental Figure 1.


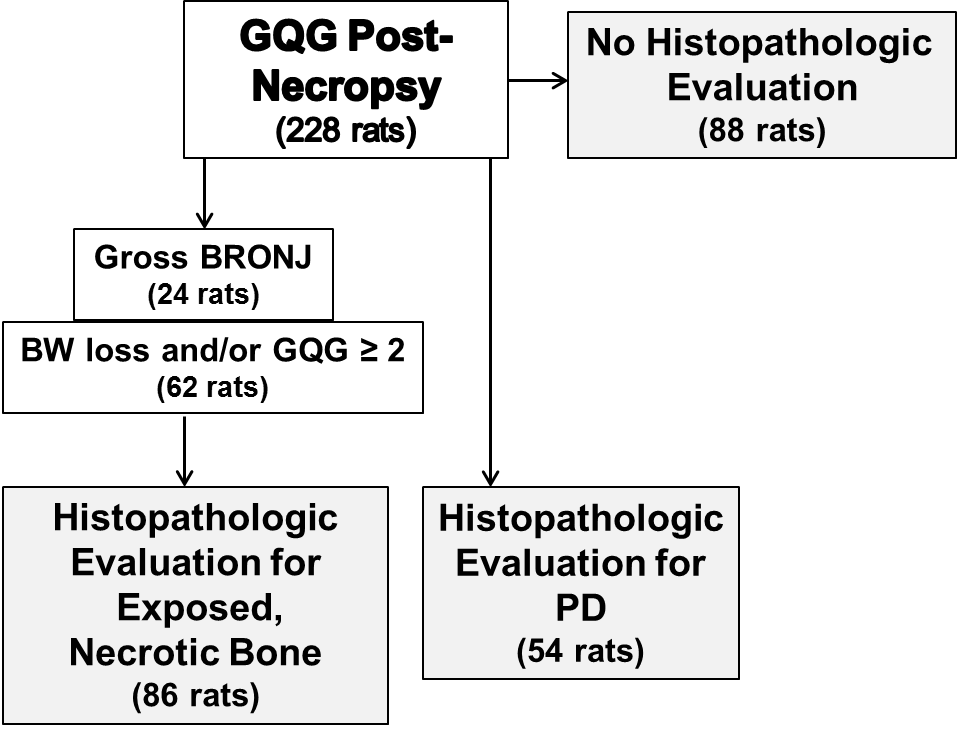


***Supporting Information 2.***

Supplemental Figure 2***
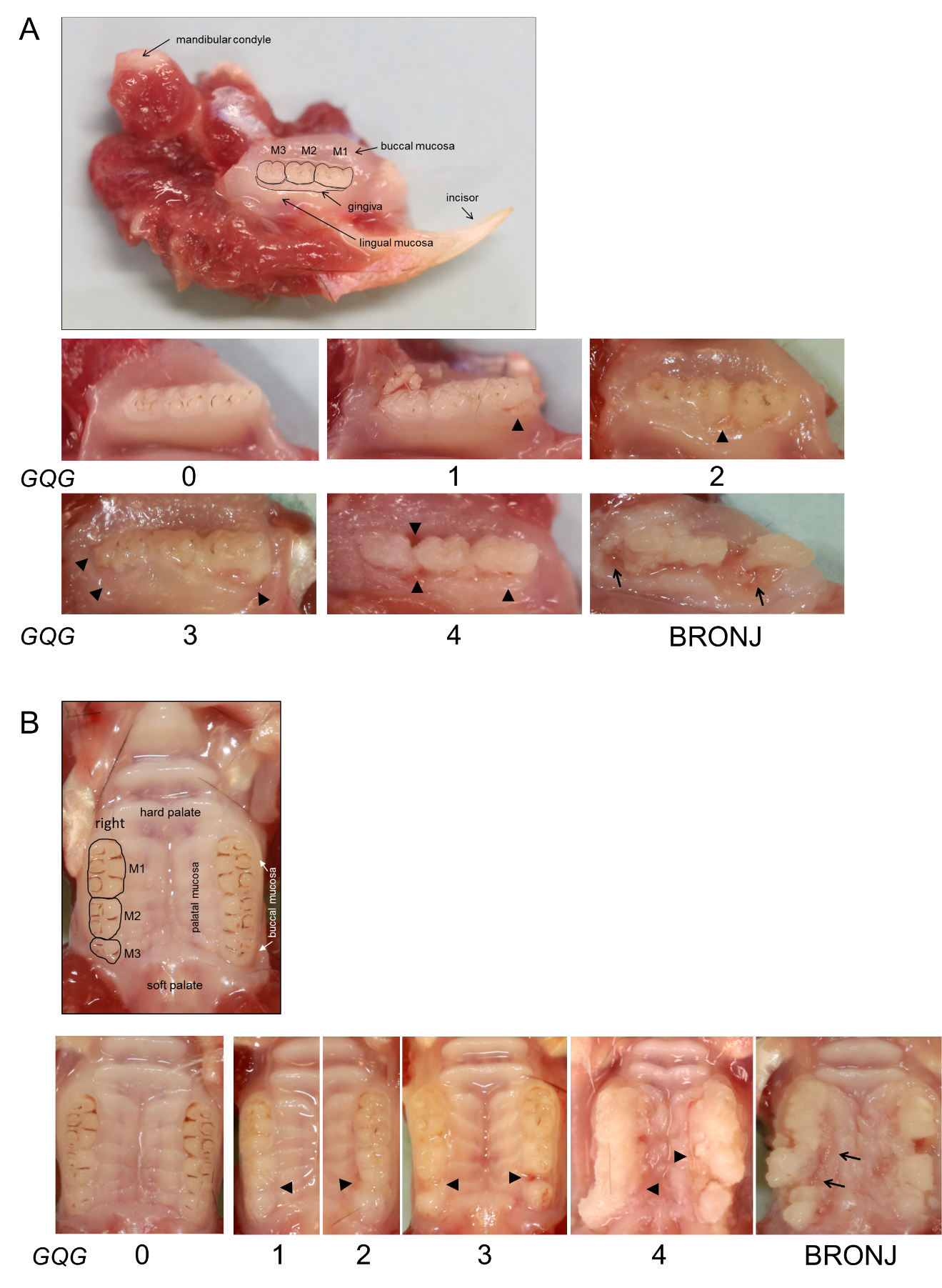
***

***Figure 2 Legend***

Representative images of A) mandibles and B) maxillae at each gross quadrant grade (*GQG*). A) Medial aspect left mandibular anatomy. *GQG*0: Intact gingiva on all aspects of all molars (M). *GQG*1: Gingival recession (arrowhead) at lingual aspect of M1. *GQG*2: Gingival recession at M1M2 interdental region and lingual aspect of M1. *GQG*3: Gingival erosion along lingual surface of M3; erosion of gingiva on lingual surface of M1, exposing furcation and cementum of M1 and extending onto mesiolingual surface of M2. *GQG*4: Severe gingival erosion along lingual and buccal aspects of M1M3, with bone loss exposing cementum of M1M3; exposed bone not apparent. *BRONJ*: Gingival erosion with loss of alveolar bone, mesial migration of M1, and exposed bone along M1M2 lingual surface and distolingual of M3 (arrows). B) Ventral view of maxillary anatomy. *GQG*0: Intact gingiva on all aspects of all molars. *GQG*1: Minimal gingival recession (arrowhead) at lingual aspect of M2M3 interdental region. *GQG*2: Gingival recession in M2M3 interdental region extending along lingual gingival margins of M2 and M3. *GQG*3: Gingival erosion/ulceration on lingual and buccal aspects of M2M3 interdental region. *GQG*4: Profound plaque accumulation with severe gingival erosion/ulceration around all molars, with malaligned teeth. *BRONJ*: Profound plaque accumulation, gingival erosion/ulceration with exposed bone along M1M3 (arrows), and buccal migration of M2 in right maxilla.

***Supplemental Figure 3***


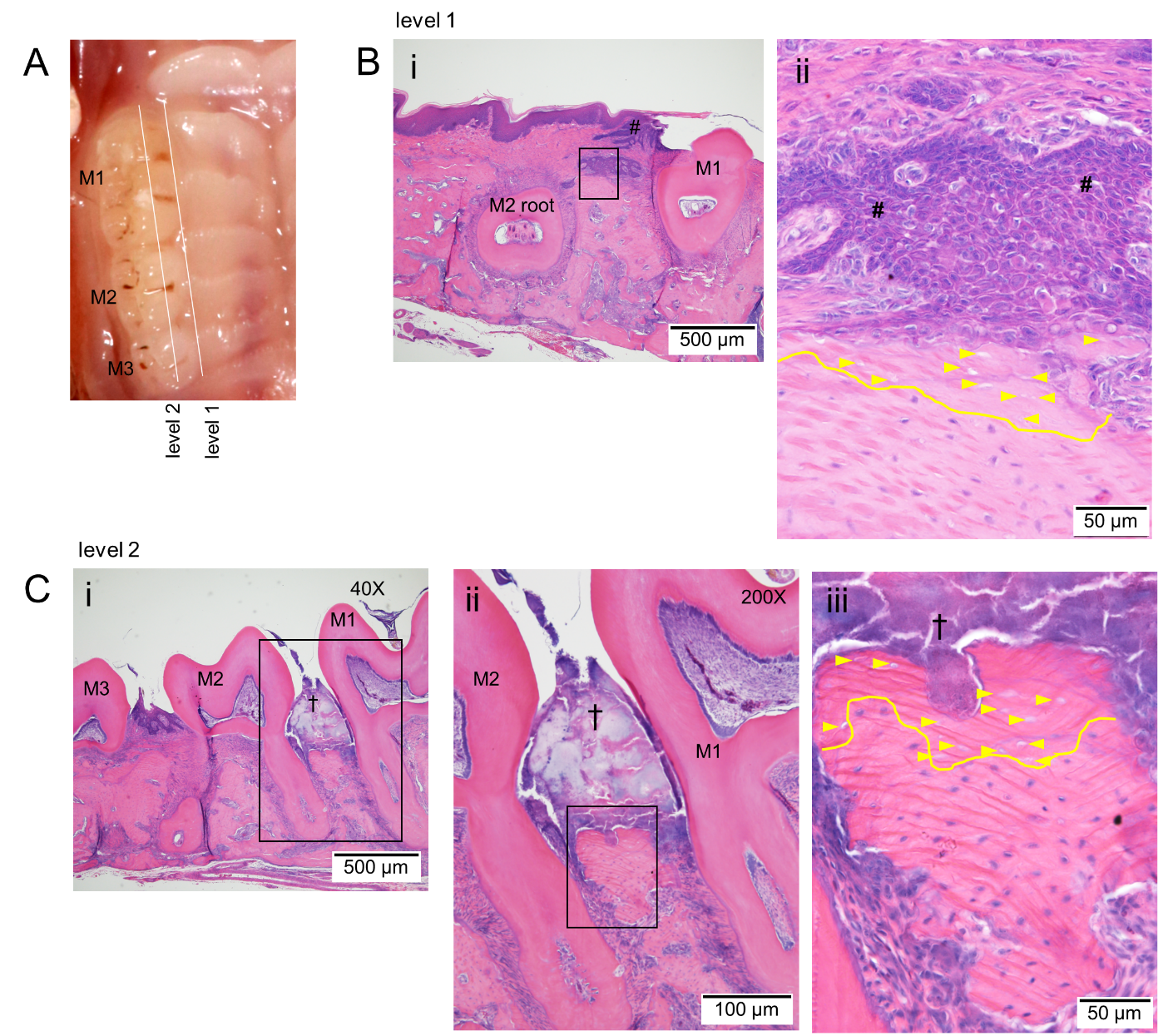


***Supporting Information 3. Histopathological analysis of BRONJ lesion with both covered and exposed necrotic bone***

***Results***

Necrotic bone was present in two levels of serial sections of a maxilla with BRONJ and from a rat that had *GQG*=0 and BW loss at necropsy (**Supplemental Figure 3A**). In the first level, necrotic bone was found underneath hyperplastic epithelial-like cells that had invaded the lamina propria and were immediately adjacent to the bone (**Supplemental Figure 3Bi and 3Bii**). In the second level, dead bone was found in the M1M2 interdental region, exposed to the oral cavity with adherent bacterial plaque (**Supplemental Figure 3Ci-3Ciii**).

***Figure 3 Legend***

Features of BRONJ lesions in two areas of a representative maxilla without gross bone exposure and *GQG*=0. A) High-resolution photograph and approximation of two levels from serial sections. Bi) section from level 1, featuring epithelial hyperplasia (#) and dead bone at the alveolar bone crest in the interdental region of molar (M)1-M2 with Bii) confluent area of empty osteocyte lacunae (yellow arrowheads) covered by epithelial-like cells. Ci) section from level 2; Cii-iii) confluent area of empty osteocyte lacunae in necrotic bone with adherent bacterial plaque (†) that is exposed to the oral cavity.
